# Effect of cardiopulmonary bypass on plasma and erythrocytes oxylipins

**DOI:** 10.1186/s12944-023-01906-z

**Published:** 2023-08-29

**Authors:** Tong Liu, Inci Dogan, Michael Rothe, Evgenij Potapov, Felix Schoenrath, Maik Gollasch, Friedrich C. Luft, Benjamin Gollasch

**Affiliations:** 1 Experimental and Clinical Research Center (ECRC), a joint institution of the Charité Medical Faculty and Max Delbrück Center (MDC) for Molecular Medicine, 13125 Berlin, Germany; 2 LIPIDOMIX GmbH, Robert-Rössle-Str. 10, 13125 Berlin, Germany; 3 Department of Cardiothoracic and Vascular Surgery, Deutsches Herzzentrum Der Charité (DHZC), Augustenburger Platz 1, 13353 Berlin, Germany; 4 DZHK (German Center for Cardiovascular Research), Partner Site Berlin, Germany Charité − Universitätsmedizin Berlin, corporate member of Freie Universität Berlin, Humboldt-Universität Zu Berlin, Potsdamer Str. 58, 10785 Berlin, Germany; 5 Department of Internal Medicine and Geriatrics, University Medicine, Greifswald, 17475 Greifswald, Germany; 6 Department of Nephrology and Internal Intensive Care, Charité – University Medicine, Augustenburger Platz 1, 13353 Berlin, Germany; 7 HELIOS Klinikum Berlin-Buch, Schwanebecker Chaussee 50, 13125 Berlin, Germany

**Keywords:** Cardiopulmonary bypass, Eicosanoids, Lipidomics, Plasma oxylipins, Erythrocyte

## Abstract

**Background:**

Oxylipins, the oxidative metabolites of polyunsaturated fatty acids (PUFAs), serve as key mediators of oxidative stress, inflammatory responses, and vasoactive reactions in vivo. Our previous work has established that hemodialysis affects both long chain fatty acids (LCFAs) and oxylipins in plasma and erythrocytes to varying degrees, which may be responsible for excess cardiovascular complications in end-stage renal disease. In this study, we aimed to determine changes in blood oxylipins during cardiopulmonary bypass (CPB) in patients undergoing cardiac surgery to identify novel biomarkers and potential metabolites of CPB-related complications. We tested the hypothesis that CPB would differentially affect plasma oxylipins and erythrocytes oxylipins.

**Methods:**

We conducted a prospective observational study of 12 patients undergoing elective cardiac surgery with expected CPB procedure. We collected venous and arterial blood samples before CPB, 15 and 45 min after the start of CPB, and 60 min after the end of CPB, respectively. Oxylipins profiling in plasma and erythrocytes was achieved using targeted HPLC‐MS mass spectrometry.

**Results:**

Our results revealed that most venous plasma diols and hydroxy- oxylipins decreased after CPB initiation, with a continuous decline until the termination of CPB. Nevertheless, no statistically significant alterations were detected in erythrocytes oxylipins at all time points.

**Conclusions:**

CPB decreases numerous diols and hydroxy oxylipins in blood plasma, whereas no changes in erythrocytes oxylipins are observed during this procedure in patients undergoing cardiac surgery. As lipid mediators primarily responsive to CPB, plasma diols and hydroxy oxylipins may serve as potential key biomarkers for CPB-related complications.

**Supplementary Information:**

The online version contains supplementary material available at 10.1186/s12944-023-01906-z

## Background

**Fig. 1:**
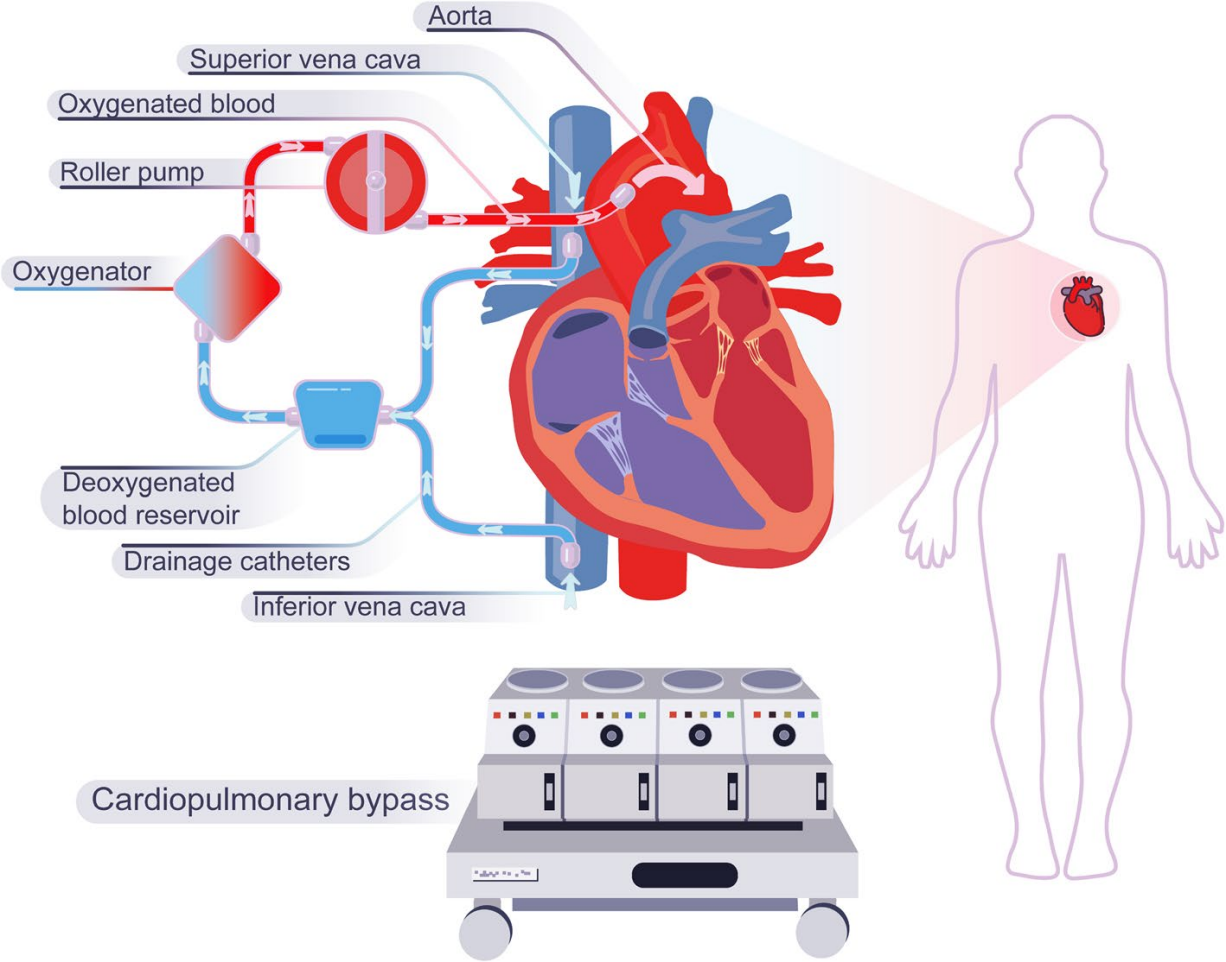
Cardiopulmonary bypass. Deoxygenated venous blood is withdrawn from the right ventricle, formed into oxygenated arterial blood by the action of the oxygenator and auxiliary devices such as roller pumps, and then returned to the aorta

Since the groundbreaking work of Gibbon et al. on the heart–lung machine in the 1950s, the cardiopulmonary bypass (CPB) has steadily advanced, allowing for comprehensive optimization of the treatment of patients with severe cardiopulmonary disease [1]. Although most cardiac operations (like heart valve surgery, aortic vascular surgery, etc.) benefit from CPB, it also results in a variety of postoperative complications. In particular, the systemic inflammatory response (SIR), activated mainly by the interaction between blood and mechanical components (Fig. 1) [1], is regarded as a leading contributor to postoperative comorbidities [2]. All organs are affected by the CPB system owing to the contact between blood and artificial material, continuous flow, hemodilution, and anticoagulation [1]. The primary comorbidities of CPB include hemolysis, bleeding, cardiac arrhythmias, respiratory failure, neurological or neuropsychiatric dysfunction, inflammation, and acute renal injury (AKI) [2, 3]. Given the physiological and pathological importance of oxylipins, particularly in inflammatory, cardiovascular, renal, and neurological diseases, and their variability in response to surgical or other stimulating conditions, the study of their variability during CPB is crucial for understanding the development of postoperative CPB comorbidities [4, 5].

**Fig. 2:**
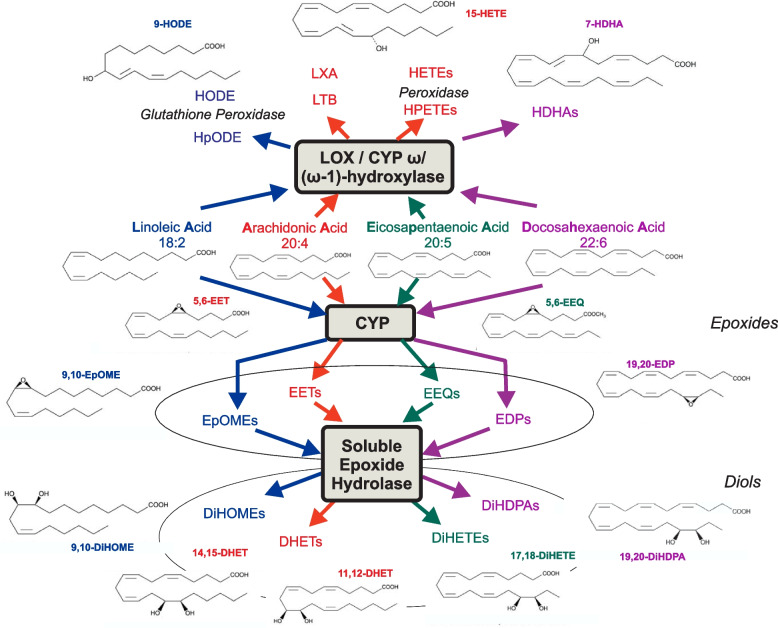
Cytochrome P450 epoxygenase (CYP) and lipoxygenase (LOX) / CYPω/(ω-1)-hydroxylase metabolize linoleic (LA), arachidonic (AA), docosahexaenoic acids (DHA), and eicosapentaenoic (EPA) in response to cardiopulmonary bypass procedure. The oxylipins detected in these metabolic pathways correspond to LA, AA, EPA, and DHA metabolism changes. AA, LA, EPA, and DHA are transformed to epoxyeicosatrienoic acid (EETs, e.g., 5,6-EET), epoxyoctadecenoic acids (EpOMEs, e.g., 9,10-EpOME), epoxyeicosatetraenoic acids (EEQs, e.g., 5,6-EEQ), and epoxydocosapentaenoic acids (EDPs, e.g., 13,14-EDP) by CYP epoxygenase, respectively. EpOMEs, EETs, EEQs, and EDPs are mainly hydrolyzed to dihydroxyctadecenoic acids (DiHOMEs, e.g., 9,10-DiHOME), dihydroxyeicosatrienoic acids (DHETs, e.g., 14,15-DHET), dihydroxyeicosatetraenoic acids (DiHETEs, e.g., 17,18-DiHETE), and dihydroxydocosapentaenoic acids (DiHDPAs, e.g., 19,20-DiHDPA) by the soluble epoxide hydrolase (sEH). LA, EPA, AA, and DHA are converted to hydroperoxylinoleic acids (HpODEs), hydroxyoctadecadienoic acids (HODEs, e.g., 9-HODE), leukotriene B (LTB), lipoxin A (LXA), hydroxydocosahexaenoic acids (HDHAs, e.g., 7-HDHA), hydroperoxyeicosatetraenoic acids (HPETEs), and hydroxyeicosatetraenoic acids (HETEs, e.g., 15-HEPE) by LOX, CYP ω/(ω-1)-hydroxylase, and peroxidase pathways. Modified from [8]

Oxylipins are primarily oxidized derivatives of n-3 and n-6 polyunsaturated fatty acids (PUFAs) formed by cyclooxygenase (COX), lipoxygenase (LOX) /CYP ω/(ω-1)-hydroxylase and cytochrome P450 (CYP450), which are widely studied for their valuable biological activities in vivo [6]. In recent years, the more studied oxylipins are the eicosanoids formed by the above three enzymes acting on arachidonic acid (AA), namely epoxyeicosatrienoic acids (EETs), dihydroxyeicosatrienoic acids (DHETs), hydroxyeicosatetraenoic acids (HETEs), and prostaglandins [7]. In addition, CYP450 and LOX/CYP ω/(ω-1)-hydroxylase also act on linoleic acid (LA), docosahexaenoic acid (DHA) and eicosapentaenoic acid (EPA) to produce other bioactive mediators such as epoxyoctadecenoic acids (EpOMEs), dihydroxyctadecenoic acids (DiHOMEs), hydroxyoctadecadienoic acids (HODEs), epoxydocosapentaenoic acids (EDPs), dihydroxydocosapentaenoic acids (DiHDPAs), hydroxydocosahexaenoic acids (HDHAs), epoxyeicosatetraenoic acids (EEQs), dihydroxyeicosatetraenoic acids (DiHETEs), and hydroperoxyeicosatetraenoic acids (HEPEs) (Fig. 2, modified from [8]). The detailed biological functions of these lipid mediators currently remain part of scientific research. In particular, the biological effects of AA-derived eicosanoids have been increasingly characterized. Eicosanoids play a role in inflammation, immune regulation, and vasoactive regulation in vital organs like the heart and kidneys [9]. As a result, they are not only physiologically important, but also are involved in systemic dysfunction and disease.

Most of the previous studies on CPB comorbidity have focused on alterations in myocardial injury markers when performing ischemia–reperfusion, neurological damage from uncontrolled reoxygenation, and AKI due to hemolysis [10–12]. Few studies have investigated how CPB affects plasma and erythrocyte oxylipins, which are considered to be vital mediators involved in oxidative stress injury, vascular endothelial damage and inflammatory responses in vivo. Recently, Kim-Campbell et al. [13] showed that plasma levels of 9-HODE and 13-HODE are elevated in children after CPB procedures and may be associated with inflammation and vasoactivity. Currently, no larger-scale lipidomic studies have been performed to evaluate the specific effects of CPB surgery on oxylipin metabolism, based on a wide literature search. In earlier studies, we established a lipidomic methodology to analyze the oxylipins in human blood. We demonstrate that bioactive endogenous n-3 and n-6 CYP lipid mediators are released by short-term maximal cardiovascular stress in peripheral blood that may affect cardiovascular function [14, 15]. We also observed that hemodialysis treatment reduced the accumulation of erythrocyte saturated fatty acids (SFAs), monounsaturated fatty acids (MUFAs), and n-6 PUFA in the peripheral tissues of patients, but promoted the release of plasma CYP 450 oxylipins into the circulating blood [8, 16, 17]. Consequently, we tested the hypothesis that the levels of plasma-oxylipins and erythrocyte-oxylipins would change during CPB and that distinct specific patterns of change would be observed.

## Methods

### Study design and patients

The ethical committee of the Charité University Medicine approved the study (EA2/311/20). Written informed consent was obtained from all participants. The study was duly registered on the ClinicalTrials.gov website (Identifier: NCT04598360). The study enrolled 12 low-risk patients undergoing elective open-heart surgery with an expected CPB at one institution. Patients with the following interventions or conditions were excluded: declined informed consent; age under 18 years, transplant surgery; emergency and urgent procedures; hemoglobin (Hb) < 8.0 g/dL; blood transfusion during surgery; pregnancy.

### Cardiopulmonary bypass 

All surgeries were performed through a median sternotomy and CPB was established with the ascending aorta and bicaval cannulations. Anesthetics used included propofol, sufentanil, and rocuronium administered intravenously. CPB was carried out with a 40 μm arterial blood filter (Terumo Capiox FX25, Germany), a pulsatile roller pump (Stockert S5, Germany) and hollow-fiber oxygenators. Moderate hypothermia was used (36 °C), and local hypothermia for myocardial protection was achieved by anterograde infusion of a cool cardioplegic solution (Cardi-Braun). Anticoagulation was achieved by administration of heparin (500 IU/kg) before the onset of CPB and was monitored by means of the activated clotting time (> 480 s). Our standard protocol for CPB was as follows: (1) flow of 2.5–3.0 L/min/body m^2^ and mean blood pressure of 50–70 mmHg; (2) rectal temperature of 36.0–37.0 °C; (3) partial pressure of oxygen (PaO2) of 200–300 mmHg, partial pressure of carbon dioxide (PaCO2) of 35–45 mmHg, and pH of 7.35–7.45 under α-stat management. After removing the aortic cross clamp and weaning from CPB, protamine was administered to obtain an activated clotting time (ACT) of whole blood of less than 140 s. All patients received standardized postoperative care.

### Blood sample collection

We collected central venous blood samples from the right subclavian or internal jugular vein placement and arterial blood samples from the radial artery. These samples had been collected at the following time points: Pre-CPB (Time point 1, P1), 15 min after CPB starts (Time point 2, P2), 45 min after CPB starts (Time point 3, P3), 60 min after CPB ends (Time point 4, P4). All blood samples were collected via 4 °C precooled EDTA vacuum extraction tube systems. The blood samples were centrifuged at 1,370 g for 10 min. Plasma and RBC were separated and stored in a freezer at -80 °C for subsequent oxylipins analysis. Clinical biochemical parameters were assessed in a qualified clinical laboratory using established procedures.

### Extraction of oxylipin profiles

For the detection of plasma or erythrocytes oxylipins, we added 300 μL of 10 M sodium hydroxide (NaOH) to a plasma (200 μL) or erythrocytes (200 μg) sample and hydrolyzed it at 60 °C for 30 min. The pH of the sample was corrected to 6 with 300 μL of 58% acetic acid. After that, solid phase extraction (SPE) was performed on the prepared samples using a Varian Bond Elut Certify II column. Specific experimental procedures were described as previously [8, 16, 18].

The extracted oxylipins were detected by LC–MS/MS using an Agilent 6460 Triple Quad mass spectrometer (Agilent Technologies, Santa Clara, CA) and an Agilent 1200 high-performance liquid chromatography (HPLC) system (degasser, binary pump, well plate sampler, thermostatic column chamber). A Phenomenex Kinetex column (150 mm 2.1 mm, 2.6 m; Phenomenex, Aschaffenburg, Germany) was used in the HPLC system. Detailed information on chromatographic analysis are provided in Fischer et al. [18] and our previous publications [8, 16]. The complete list of detected oxylipins and their corresponding parameters for multiple reaction monitoring are provided in Table S1. Internal standards (ISTD) added to the samples consisted of 10 ng each of 20-HETE-d6, 9,10-DiHOME-d4, 12,13-EpOME-d4, 13-HODE-d4, 8,9-EET-d11, and 15-HETE-d8 (Cayman Chemical) for quantification of similar oxylipin clusters. The lower limit of quantification (LOQ) was 0.01 ng/ml or 0.1 ng/g. Quantitative calibration curves for individual oxylipins were built according to the variation of the relative peak areas of different target compound/ISTD concentration ratios. The linearity was r^2^ > 0.99 for any compound in the absolute value range of 1 to 20 ng.

### Statistical analysis

The obtained data were subjected to descriptive statistics, and the variables' skewness and kurtosis were checked to see if they conformed with the assumption of normal distribution. If the data were normally distributed, the four time points were compared using one-way repeated measures analysis of variance (ANOVA). The analysis included Mauchly's test of sphericity followed by applying the test of within‐subjects' effects with Greenhouse–Geisser correction, which is used for repeated‐measures ANOVA when the assumption of sphericity is violated. The nonparametric test (Friedman’ s test) was used when the normal distribution was violated. The Tukey’ s method was used as a post hoc two-by-two comparison. A *p*-value less than 0.05 was regarded as statistically significant. All data are presented as mean ± standard deviation (SD). SPSS Statistics software was used to execute the statistical analysis (IBM Corporation, Armonk, NY, USA). MetaboAnalyst 5.0 software (https://www.metaboanalyst.ca/) was used for generating heatmaps.

## Results

### Participant characteristics and clinical parameters

The mean age of participants was 71 years, 5/12 (42%) participants were male, and 7/12 (58%) participants were female. The majority of participants were undergoing minimal invasive mitral valve surgery (5/12; 42%), minimal invasive tricuspid valve surgery (2/12; 17%), coronary artery bypass grafting (2/12; 17%), or aortic valve and vascular surgery (3/12, 25%) (Table 1). Basic clinical parameters of the participants are given in the supplement (Table S2**)**, and comparing the parameters at all four-time points did not identify statistically significant differences. The average duration of CPB for all patients was 75 min.

**Table 1:** Patients characteristics (*n* = 12)

	**Patients**
Age (years)	71 ± 8
Sex	
Male (*n*)	5/12 (42%)
Female (*n*)	7/12 (58%)
Body mass index (kg/m^2^)	26 ± 7
**Diagnoses/surgery**	
Minimal invasive mitral valve surgery	5
Minimal invasive tricuspid valve surgery	2
Coronary artery bypass graft	2
Valve-sparing aortic root replacement	1
Annuloaortic ectasia	1
Aortic valve surgery	1

Data presented as mean ± standard deviation, n (%)

### CYP 450-epoxy and LOX/CYP ω/(ω-1)-hydroxylase oxylipins in venous plasma and erythrocytes

**Fig. 3:**
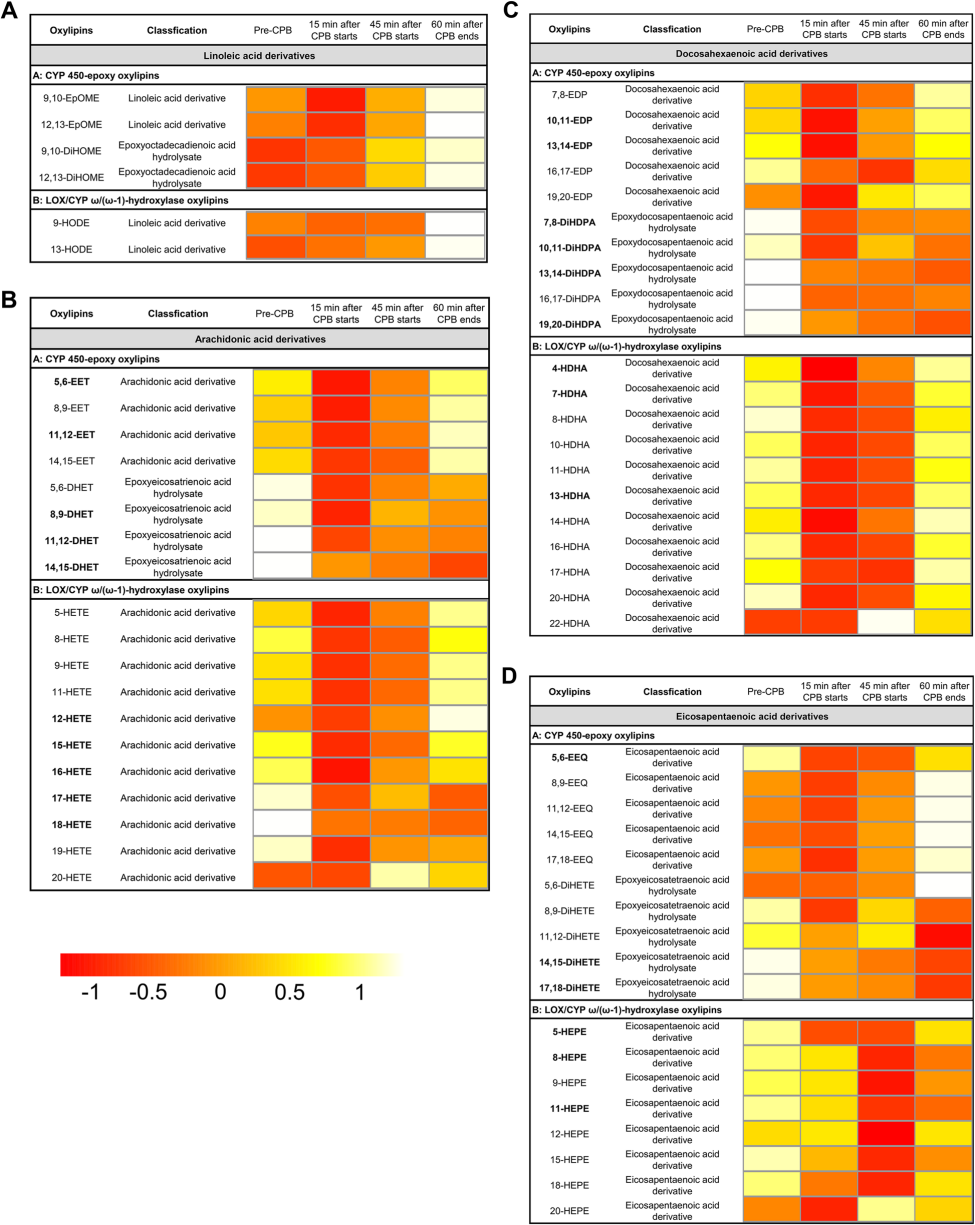
Heatmap analysis of venous plasma oxylipins. Heatmap shows the average oxylipin levels in each group. **A** LA-derived oxylipins. **B** AA-derived oxylipins. **C** DHA-derived oxylipins. **D** EPA-derived oxylipins. The color from red to white in the heatmap reflects the gradually increasing concentration of oxylipins

As the effect of CPB on circulating levels of CYP and LOX mediators is unknown, we took an exploratory statistical method for the measured CYP and LOX-dependent oxylipins. Firstly, we measured the oxylipins in plasma at four different time points: Pre-CPB, 15 min after CPB starts, 45 min after CPB starts, 60 min after CPB ends. Our ANOVA results revealed that the majority of oxylipins showed statistical differences between the four time points (Fig. 3, Table S3). We found that LA-derived oxylipins in plasma showed no differences (Fig. 3A, Table S3). However, the majority of the plasma oxylipins derived from AA showed significant changes. These oxylipins included 5, 6-EET, 11, 12-EET, 8, 9-DHET, 11, 12-DHET, 14, 15-DHET, 12-EHTE, 15-HETE, 16-HETE, 17-HETE, and 18-HETE (Fig. 3B, Table S3). We also observed changes in DHA-derived oxylipins in plasma, which included 10, 11-EDP, 13, 14-EDP, 7, 8-DiHDPA, 10, 11-DiHDPA, 13, 14-DiHDPA, 19, 20-DiHDPA, 4-HDHA, 7-HDHA, and 13-HDHA (Fig. 3C, Table S3). A few oxylipins derived from EPA in plasma also displayed statistically significant changes. These oxylipins included 5, 6 EEQ, 14, 15-DiHETE, 17, 18-DiHETE, 5-HEPE, 8-HEPE and 11-HEPE (Fig. 3D, Table S3). To better understand the changes, we performed a two-by-two comparison of the oxylipins at different time points (Table S4). We observed that the levels of most oxylipins declined significantly during the CPB run (P2, P3) compared to before CPB initiation (P1). These oxylipins were 8,9-DHET, 11,12-DHET, 14,15-DHET, 13,14-EDP, 7,8-DiHDPA, 10,11-DiHDPA, 13,14-DiHDPA, 19,20-DiHDPA, 5,6-EEQ, 17,18-DiHETE, 15-HETE, 16-HETE, 17-HETE, 18-HETE, 4-HDHA, 5-HEPE, and 11-HEPE. Interestingly, there was small amounts of oxylipin levels that were the first to rebound at 60 min after CPB ends (P4) compared to during CPB proceeds (P2, P3). These oxylipins were 11,12-EET, 10,11-EDP, 13,14-EDP, 12-HETE, and 4-HDHA.

**Fig. 4:**
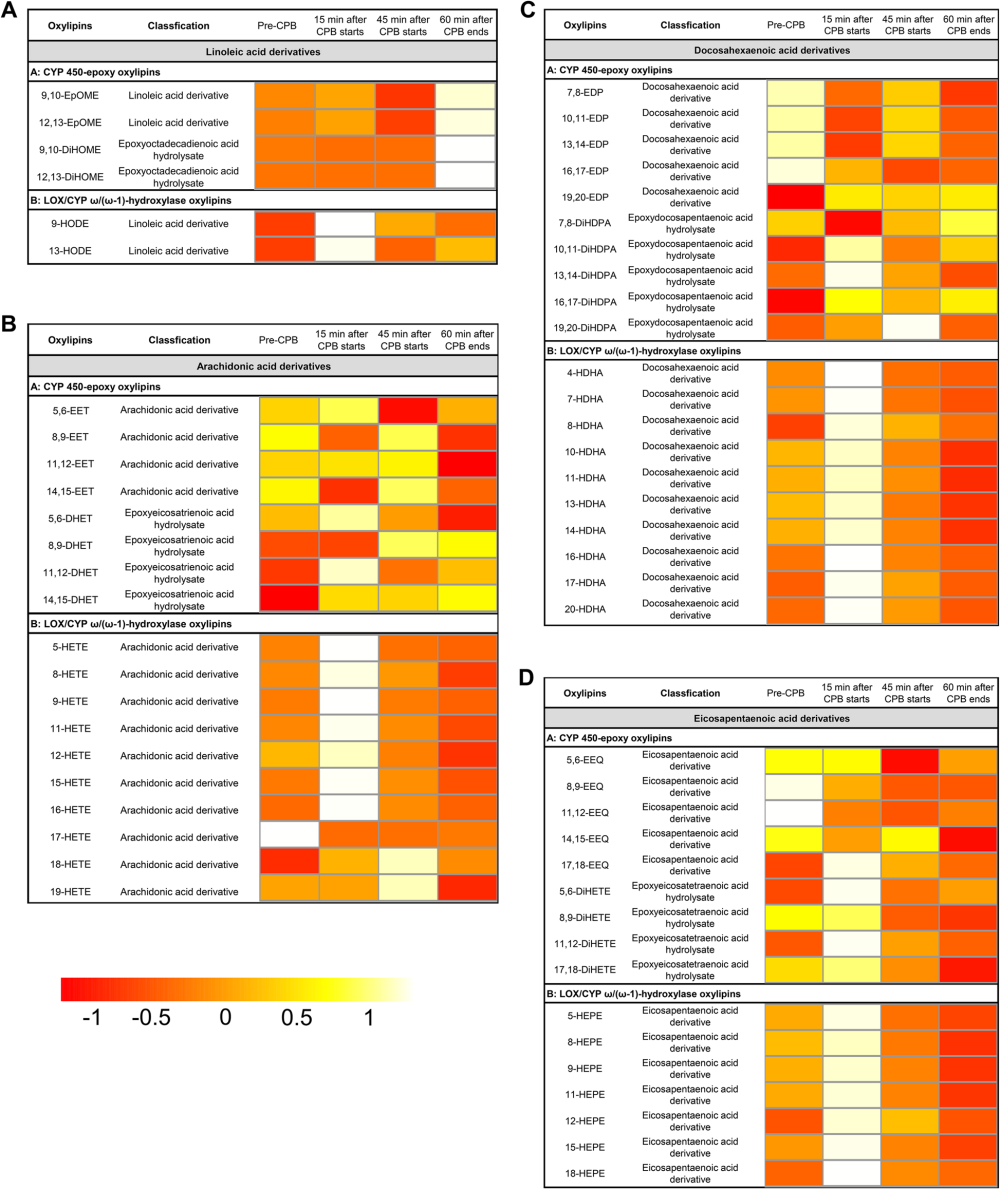
Heatmap analysis of venous erythrocytes oxylipins. Heatmap shows the average oxylipin levels in each group. **A** LA-derived oxylipins. **B** AA-derived oxylipins. **C** DHA-derived oxylipins. **D** EPA-derived oxylipins. The color from red to white in the heatmap reflects the gradually increasing concentration of oxylipins

Next, oxylipins in erythrocytes were measured at the four different time points. In contrast to the changes in plasma oxylipins, the ANOVA showed no statistical differences in erythrocytes oxylipins (Fig. 4, Table S5). This finding indicated that the erythrocyte oxylipins would remain stable in the CPB surgical state.

### CYP 450-epoxy and LOX/CYP ω/(ω-1)-hydroxylase oxylipins in arterial plasma and erythrocytes

**Fig. 5:**
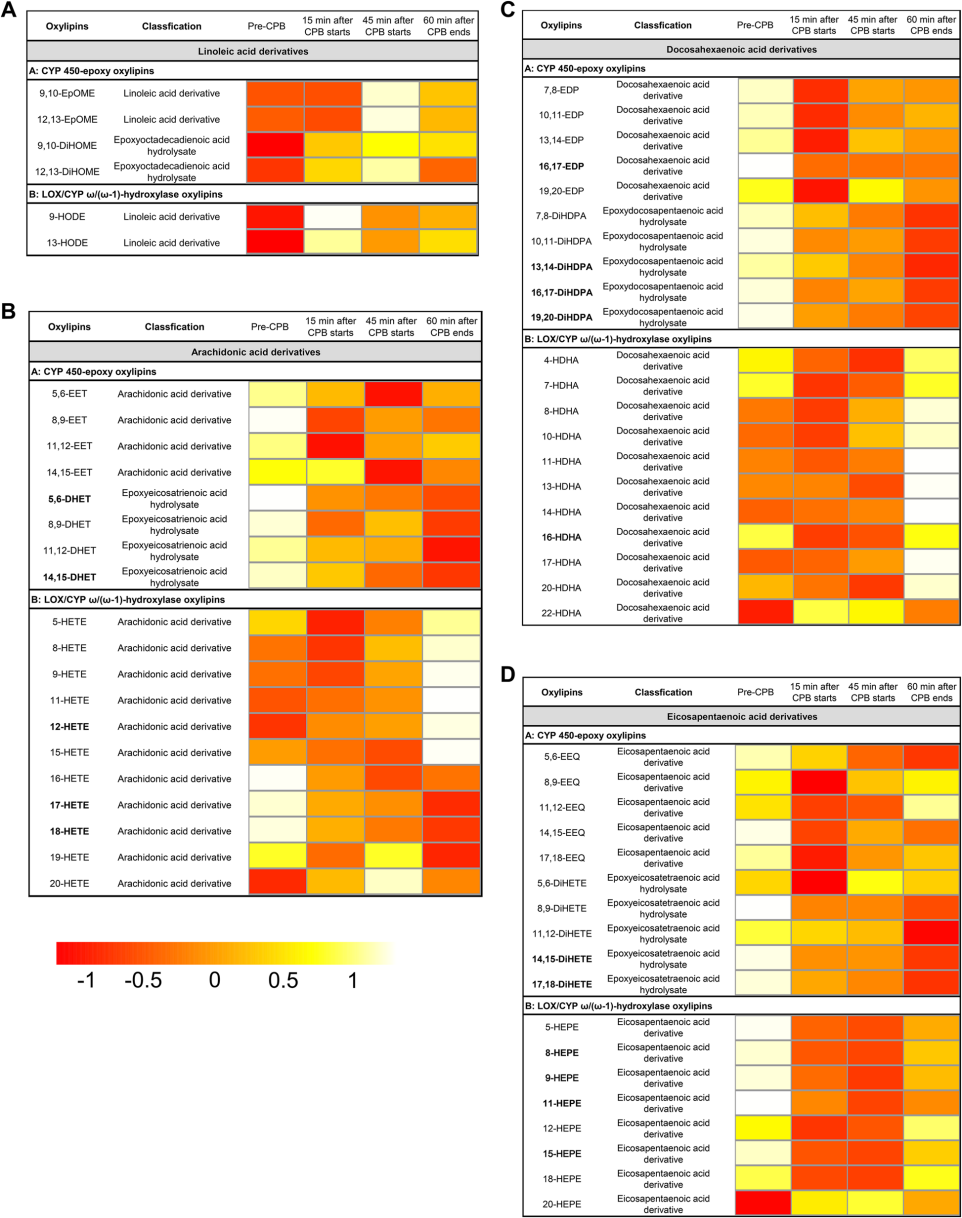
Heatmap analysis of arterial plasma oxylipins. Heatmap shows the average oxylipin levels in each group. **A** LA-derived oxylipins. **B** AA-derived oxylipins. **C** DHA-derived oxylipins. **D** EPA-derived oxylipins. The color from red to white in the heatmap reflects the gradually increasing concentration of oxylipins

To further validate the pattern of CPB effects on CYP 450- and LOX/CYP ω/(ω-1)-hydroxylase oxylipins, we also assayed the changes in these oxylipins in arterial plasma and erythrocytes. ANOVA-test revealed that most arterial plasma oxylipin levels also showed statistical differences at the four different time points, broadly consistent with the changing pattern of central venous plasma oxylipins (Fig. 5, Table S6). Significant changes were observed for the AA-derived oxylipins, 5,6-DHET, 14,15-DHET, 12-HETE, 17-HETE, and 18-HETE (Fig. 5B, Table S6), the DHA-derived oxylipins, 16,17-EDP, 13,14-DiHDPA, 16,17-DiHDPA, 19,20-DiHDPA, and 16-HDHA (Fig. 5C, Table S6), and the EPA-derived oxylipins, 14,15-DiHETE, 17,18-DiHETE, 8-HEPE, 9-HEPE, 11-HEPE, and 15-HEPE (Fig. 5D, Table S6). All of these oxylipins manifested significantly lower levels during CPB proceeds (P2, P3) than their preoperative levels (P1) in a two-by-two post hoc comparison (Table S7).

In arterial erythrocytes we identified only a few oxylipins that presented statistical differences in the ANOVA test. These oxylipins included 11,12-DHET, 19,20-DiHDPA, 9-HODE, 13-HODE, 9-HETE, 4-HDHA, and 9-HEPE (Table S8). Two-by-two post hoc comparisons revealed that the levels of these oxylipins were higher during the CPB proceeding (P2, P3) than the levels at 60 min after the CPB ends (P4) (Table S9). Taken together, the majority of erythrocyte oxylipin levels remained unchanged in response to CPB manipulation and stimulation.

## Discussion

Our study is the first large-scale targeted lipidomic study of cardiac patients undergoing CPB. This study aimed to map the lipid mediator profiles in plasma and in RBCs of patients with elective cardiac disease undergoing CPB. In particular, we aimed to determine the specific effects of the CPB procedure on oxylipins in both plasma and red blood cells to identify novel biomarkers that may be responsible for CPB-related complications. We found that the levels of plasma diols and hydroxy-oxylipins at baseline are highest in patients undergoing elective cardiac surgery, and that CPB decreased these oxylipins. We found that total oxylipins in erythrocyte remain stable in response to CPB.

CPB is performed by withdrawing venous blood from the right ventricle through a catheter, transforming it into oxygenated arterial blood by different CPB auxiliary systems (traps, clamps, humidifiers, heat exchanger, etc.) and oxygenators, and then returning it to the aorta for body circulation (Fig. 1) [19]. According to recent research [20, 21], blood exposure to artificial tubes may stimulate an inflammatory response, activate the coagulation system, and activate bioactive factors, which might be closely related to postoperative CPB complications. Eicosanoids, such as prostaglandins (PGs), leukotrienes (LTs), HETEs, and EETs, play important roles in inflammatory responses at organ and tissue level [22–25]. In particular, epoxy oxylipins are rapidly transformed into dihydroxy derivatives by soluble epoxide hydrolase (sEH), such as DHETs, DiHETEs, and DiHDPAs, which were initially believed to be biologically inactive but are now also thought to have unique biological features [26]. For example, DHETs exhibit more potent vasodilatory effects than EETs in mouse, rat and canine models [27, 28]. DiHDPAs inhibit platelet aggregation in humans and reduce inflammation-related pain more effectively than EDP and EET [29, 30]. DiHETEs could inhibit human platelet aggregation but not as effectively as EEQs [7]. HETEs also have unique biological activities in vivo, such as stimulation of cytokine release, stimulation of leukocyte migration, potent vasoconstrictor effects and activation of peroxisome proliferator-activated receptors (PPAR) [31, 32]. 7-HDHA is an endogenous, selective PPARα ligand, which promotes dendritic outgrowth and arborization associated with synaptic plasticity [33]. Presently, the impact of oxylipins alterations during CPB on the complications that occur in the organism after CPB, such as inflammatory response, pulmonary hypertension, and coagulation abnormalities, have received limited attention so far.

Our previous studies have revealed that hemodialysis procedure increases plasma epoxide oxylipins as well as decreased erythrocyte hydroxy oxylipins [8, 16]. While the exact mechanism is unclear, we concluded that extracorporeal circulation can redistribute fatty acid oxylipins in vivo. In our present study, we similarly identified that numerous plasma oxylipins levels decreased during CBP, likely reflecting a reduced formation and bioavailability of most oxylipins in plasma, but no change in RBCs. Although the specific results (i.e. individually affected oxylipins) should be interpreted with caution, the dynamic response of plasma fatty acid oxylipins in blood to this invasive manipulation is evident. William et al. [34–36] studied changes in the stable breakdown metabolites (thromboxane B2 and 6-keto-PGF1 alpha) of thromboxane and prostacyclin in pediatric and adult cardiac patients undergoing CPB, respectively, and found that CPB increased both plasma thromboxane B2 and 6-keto-PGF1 alpha, although the pathophysiological implications remain uncertain. Strassburg et al. [37] found that plasma 12-HETE and 12-HEPE were elevated after cardiac surgery compared to preoperative levels. Interestingly, we detected a parallel pattern with total plasma 12-HETE levels being elevated after CPB compared to baseline (Pre-CPB). However, total plasma 12-HEPE levels were not significantly altered at the four-time points. Taken together, we conducted targeted studies on a wide range of fatty acid oxylipins for a better understanding of CPB manipulation effects on these oxylipins in the present study. We were able to demonstrate that following the onset of CPB, a notable reduction in circulating venous plasma concentrations of diols and hydroxy-oxylipins was observed, and this decline persisted until the cessation of CPB. In parallel, we also analyzed changes in arterial plasma for the above oxylipins and detected a similar pattern of changes (decrease after CPB onset), which provides evidence for a temporal correlation between these lipid mediators and CPB. The specific mechanisms that led to our observations are not yet clear, but likely to be multifactorial in origin, and may particularly depend oxygen availability. However, as we observed steady state physiological hyperoxic conditions throughout our protocol (reflected in blood gas analysis, Table S2), we assume that CPB per se, which is known to cause oxidative stress, extracorporeal circulation, red blood cell-endothelium interactions, chronic inflammation, heparin induced lipoproteases etc., appears to be an independent contributor for alterations in oxylipin profiles rather than oxygen availability in particular [38]. When exposed to hypoxia or other environmental stimuli, erythrocytes, which have been proven to be a reservoir of EETs, release EETs into the plasma to exert biologically active effects [39, 40]. Additionally, erythrocyte EETs directly interact with the vascular endothelium to induce vasoactive effects [27]. Our study additionally assessed the influence of CPB manipulation on erythrocyte oxylipin levels, which showed no significant changes, suggesting that erythrocyte oxylipins remain comparatively stable during CPB. This is in line with our previous findings [16], which indicated that there is little influence on the total erythrocytes oxylipin status by hemodialysis. Our study demonstrates that total erythrocyte fatty acid changes consistently show superior resilience in two distinct extracorporeal circulation operations (i.e., hemodialysis and CPB).

The present study also has limitations. First, due to financial and temporal restrictions, we were not able to extend the postoperative observation period and continuously follow the changes in oxylipin levels in patients at different time points after surgery, thus providing more clarity on the potential clinical events associated with these oxylipins. Second, the small sample size of this study only enabled us to observe which oxylipins were influenced by the CPB. However, we are not yet able to assess whether such an effect is beneficial or detrimental, so that these problems will be part of future studies.

## Conclusions

CPB operation reduced numerous plasma diols and hydroxy oxylipins of long-chain PUFAs, possibly related to blood exposure to the CPB circuit system, resulting in reduced bioavailability of these oxylipins, but this should be interpreted with caution. Conversely, erythrocyte oxylipins displayed a degree of resistance to CPB operation. Further studies are needed to determine whether these changes in oxylipins caused by CPB are deleterious or beneficial to the organism.

### Supplementary Information

**Additional file 1: Table S1.** HPLC-MS/MS conditions for the measurement of LA, AA, EPA, and DHA-derived oxylipins. **Table S2.** Descriptive statistics of clinical parameters at four different time points (P). **Table S3.** Time-profiles throughout cardiopulmonary bypass surgery with changes in expression levels of venous plasma oxylipins. **Table S4.** Post-hoc comparison of paired differences in venous plasma oxylipin levels. **Table S5.** Time-profiles throughout cardiopulmonary bypass surgery with changes in expression levels of venous erythrocytes oxylipins. **Table S6.** Time-profiles throughout cardiopulmonary bypass surgery with changes in expression levels of arterial plasma oxylipins. **Table S7.** Post-hoc comparison of paired differences in arterial plasma oxylipin levels. **Table S8.** Time-profiles throughout cardiopulmonary bypass surgery with changes in expression levels of arterial erythrocytes oxylipins. **Table S9.** Post-hoc comparison of paired differences in arterial erythrocyte oxylipin levels.

## Data Availability

The datasets used and/or analysed during the current study are available from the corresponding author on reasonable request.

## Abbreviations

PUFAs: Polyunsaturated fatty acids
LOQ: Limit of quantification
ISTD: Internal standards
SFA: Saturated fatty acids
MUFA: Monounsaturated fatty acids
LCFAs: Long chain fatty acids
CPB: Cardiopulmonary bypass
SIR: Systemic inflammatory response
AKI: Acute renal injury
COX: Cyclooxygenase
LOX: Lipoxygenase
CYP450: Cytochrome P450
AA: Arachidonic acid
EETs: Epoxyeicosatrienoic acids
DHETs: Dihydroxyeicosatrienoic acids
HETEs: Hydroxyeicosatetraenoic
LA: Linoleic acid
DHA: Docosahexaenoic acid
EPA: Eicosapentaenoic acid
EpOMEs: Epoxyoctadecenoic acids
DiHOMEs: Dihydroxyctadecenoic acids
HODEs: Hydroxyoctadecadienoic acids
EDPs: Epoxydocosapentaenoic acids
DiHDPAs: Dihydroxydocosapentaenoic acids
HDHAs: Hydroxydocosahexaenoic acids
EEQs: Epoxyeicosatetraenoic acids
DiHETEs: Dihydroxyeicosatetraenoic acids
HEPEs: Hydroperoxyeicosatetraenoic acids
PaO2: Partial pressure of oxygen
PaCO2: Partial pressure of carbon dioxide
ACT: Activated clotting time
NaOH: Sodium hydroxide
SPE: Solid phase extraction
sEH: Soluble epoxide hydrolase
PPAR: Peroxisome proliferator-activated receptors
