# Supplementary material for: Effect of cardiopulmonary bypass on plasma and erythrocytes oxylipins
Source: Lipids Health Dis. 2023 Aug 29;22:138. doi: 10.1186/s12944-023-01906-z (PMC10463967; doi:10.1186/s12944-023-01906-z)
Supplement: Supplementary file 1 — Additional file 1: Table S1. HPLC-MS/MS conditions for the measurement of LA, AA, EPA, and DHA-derived oxylipins. Table S2. Descriptive statistics of clinical parameters at four different time points (P). Table S3. Time-profiles throughout cardiopulmonary bypass surgery with changes in expression levels of venous plasma oxylipins. Table S4. Post-hoc comparison of paired differences in venous plasma oxylipin levels. Table S5. Time-profiles throughout cardiopulmonary bypass surgery with changes in expression levels of venous erythrocytes oxylipins. Table S6. Time-profiles throughout cardiopulmonary bypass surgery with changes in expression levels of arterial plasma oxylipins. Table S7. Post-hoc comparison of paired differences in arterial plasma oxylipin levels. Table S8. Time-profiles throughout cardiopulmonary bypass surgery with changes in expression levels of arterial erythrocytes oxylipins. Table S9. Post-hoc comparison of paired differences in arterial erythrocyte oxylipin levels. [file 12944_2023_1906_MOESM1_ESM.pdf]

Table S1 HPLC-MS/MS conditions for the measurement of LA, AA, EPA, and DHA-derived oxylipins.

Compound Name	ISTD	Precursor Ion [m/z]	Product Ion [m/z]	Collision Energy [V]	Fragmentor Voltage [V]	Cell Accelerator Voltage [V]	Retension Time [min]
10-HDHA	1	343.2	153.1	14	166	2	16.41
11,12-EEQ	1	317.2	179.1	11	166	2	16.37
11-HDHA	1	343.2	121.1	13	166	1	16.70
11-HETE	1	319.2	167.1	14	166	1	16.32
12-HETE	1	319.2	179.2	13	166	1	16.64
13-HDHA	1	343.2	193.2	12	166	1	16.24
14,15-EEQ	1	317.2	207.1	11	166	1	16.25
14-HDHA	1	343.2	161.1	13	166	1	16.42
15-HETE	1	319.2	219.2	11	166	1	15.95
16-HDHA	1	343.2	233.2	11	166	2	16.02
17,18-EEQ	1	317.2	259.2	10	166	2	15.73

17-HDHA	1	343.2	245.2	10	166	2	16.14
20-HDHA	1	343.2	241.2	11	166	1	15.74
5-HEPE	1	317.2	115.1	11	166	1	15.17
8,9-EEQ	1	317.2	155.1	9	166	2	16.51
8-HDHA	1	343.2	189.1	11	166	2	17.03
8-HETE	1	319.2	155.1	12	166	1	16.61
15-HETE-d8	ISTD-1	343.2	153.1	14	166	2	15.99
19-HETE	2	319.2	275.2	16	166	1	14.58
20-HETE-d6	ISTD-2	319.2	275.2	16	166	1	14.18
10,11-EDP	3	343.2	153.1	11	166	1	18.15
11,12-EET	3	319.2	167.1	12	166	1	18.27
13,14-EDP	3	343.2	234.2	7	166	1	18.04
14,15-EET	3	319.2	219.2	9	166	1	17.67
16,17-EDP	3	343.2	201.1	9	166	1	17.96
5,6-EEQ	3	317.2	189.1	9	166	1	16.49

5,6-EET	3	319.2	191.2	7	166	1	18.48
7,8-EDP	3	343.2	109	10	166	1	18.39
8,9-DiHETE	3	335.2	185.2	21	166	1	11.80
8,9-EET	3	319.2	127.1	15	166	1	18.47
8,9-EET-d11	ISTD-3	343.2	153.1	11	166	1	17.85
10,11-DiHDPA	4	361.2	153.1	16	166	1	13.88
11,12-DHET	4	337.2	167.2	18	166	1	13.52
11,12-DiHETE	4	335.2	167.1	16	166	1	11.47
12,13-DiHOME	4	313.2	183.2	20	166	1	11.94
13,14-DiHDPA	4	361.2	193.2	16	166	1	13.61
14,15-DHET	4	337.2	207.2	17	166	1	12.89
14,15-DiHETE	4	335.2	207.1	18	166	1	11.30
16,17-DiHDPA	4	361.2	233.2	15	166	1	13.42
17,18-DiHETE	4	335.2	247.2	17	166	1	10.87
18-HEPE	4	317.2	259.2	9	166	1	13.89

19,20-DiHDPA	4	361.2	273.3	15	166	1	13.04
20-HEPE	4	317.2	287.2	14	166	1	13.59
20-HETE	4	319.2	289.2	19	166	1	14.77
5,6-DiHETE	4	335.2	145	16	166	1	12.63
8,9-DHET	4	337.2	127	22	166	1	14.04
9,10-DiHOME	4	313.2	201.1	21	166	1	12.30
9,10-DiHOME-d4	ISTD-4	361.2	153.1	16	166	1	13.75
12,13-EpOME	5	295.3	195.2	16	166	1	17.52
19,20-EDP	5	343.2	285.2	7	166	1	17.49
4-HDHA	5	343.2	101	13	166	1	17.66
5-HETE	5	319.2	115.1	14	166	1	17.19
7-HDHA	5	343.2	141.1	11	166	1	16.79
9,10-EpOME	5	295.3	171.2	16	166	1	17.73
9-HETE	5	319.2	151.1	13	166	1	16.95
12,13-EpOME-d4	ISTD-5	295.3	195.2	16	166	1	17.53

11-HEPE	6	317.2	167	7	166	2	14.44
12-HEPE	6	317.2	179.1	13	166	1	14.74
13-HODE	6	295.2	195.2	18	166	1	15.52
15-HEPE	6	317.2	219.2	11	166	1	14.37
16-HETE	6	319.2	233.2	14	166	1	15.25
17-HETE	6	319.2	247.2	14	166	2	15.05
18-HETE	6	319.2	261.2	16	166	1	14.94
22-HDHA	6	343.2	269.2	13	166	1	15.48
5,6-DHET	6	337.2	145.1	16	166	1	14.89
7,8-DiHDPA	6	361.2	113.1	18	166	1	14.57
8-HEPE	6	317.2	155.1	13	166	1	14.57
9-HEPE	6	317.2	167.1	13	166	1	14.90
9-HODE	6	295.1	171	21	166	2	15.54
13-HODE-d4	ISTD-6	317.2	179.1	13	166	1	14.31

Note: ISTD: internal standard

Table S2 Descriptive statistics of clinical parameters at four different time points (P).

Parameters	Time point (P) 1: Pre-CPB	Time point (P) 2: 15 min after CPB starts	Time point (P) 3: 45 min after CPB starts	Time point (P) 4: 60 min after CPB ends	Mauchly's test/Greenhouse–Geisser, P value
**Heart rate (beats/min)**	58 ± 12	n.d	n.d	n.d	
**systolic blood pressure (mmHg)**	111 ± 22	n.d	n.d	n.d	
**diastolic blood pressure (mmHg)**	58 ± 10	n.d	n.d	n.d	
**PaO_2 (mmHg)**	409.08 ± 72.77	332.92 ± 98.56	276.58 ± 74.30	219.31 ± 98.04	
**$PaCO_2$ (mmHg)**	42.28 ± 7.39	43.35 ± 8.53	43.26 ± 6.28	41.93 ± 8.23	>0.05
**HCO_3^- (mmol/L)**	25.31 ± 1.87	25.55 ± 3.36	26.03 ± 2.44	24.49 ± 2.13	
**pH**	7.4 ± 0.06	7.36 ± 0.08	7.39 ± 0.06	7.38 ± 0.08	
**Hb (g/dL)**	11.47 ± 1.41	10.52 ± 1.38	10.72 ± 1.37	11.73 ± 1.48	
**Lactate (mg/dL)**	5.67 ±1.56	6.17 ±2.12	6.33 ±1.78	13.50 ±15.02	
**Glucose (mg/dL)**	104 ±20.2	113 ±25.77	119.42 ±29.38	125.08 ±23.91	

Data presented as Mean ±SD. PaO_2: arterial partial pressure of oxygen; $PaCO_2$: arterial partial pressure of carbon dioxide; Hb: hemoglobin; HCO_3^- : hydrocarbonate.

Table S3 Time-profiles throughout cardiopulmonary bypass surgery with changes in expression levels of venous plasma oxylipins.

Units ng/ml	Time point 1: Pre-CPB	Time point 2: 15 min after CPB starts	Time point 3: 45 min after CPB starts	Time point 4: 60 min after CPB ends	Mauchly's test/Greenhouse–Geisser, P value
**A: CYP 450-epoxy oxylipins**					
9,10-EpOME	8.39 ±2.13	7.83 ±2.46	8.49 ±2.77	9.19 ±3.11	0.29
12,13-EpOME	8.46 ±1.94	7.98 ±2	8.67 ±2.6	9.67 ±3	0.47
9,10-DiHOME	19.01 ±18.8	19.76 ±9.19	22.39 ±11.76	24.52 ±13.96	0.17
12,13-DiHOME	17.68 ±14.82	18.06 ±8.16	19.47 ±9.95	21.01 ±11.49	0.34
**5,6-EET**	**0.03 ±0.01**	**0.02 ±0.02**	**0.03 ±0.02**	**0.03 ±0.01**	**Friedman p=0.02***
8,9-EET	3.85 ± 1.37	3.33 ± 1.43	3.65 ± 1.30	4.16 ± 1.73	p=0.08
**11,12-EET**	**2 ± 0.92**	**1.66 ± 0.81**	**1.84 ± 0.69**	**2.26 ± 1.15**	**p=0.02***
14,15-EET	5.83 ± 1.96	5.02 ± 2.47	5.21 ± 1.89	6.3 ± 2.54	p=0.13
5,6-DHET	1.13 ±0.72	0.91 ±0.39	0.98 ±0.48	1.01 ±0.62	0.55
**8,9-DHET**	**1.46 ±0.54**	**1.18 ±0.42**	**1.32 ±0.57**	**1.29 ±0.59**	**Friedman p=0.00*** **
**11,12-DHET**	**0.58 ±0.19**	**0.46 ±0.15**	**0.49 ±0.19**	**0.49 ±0.19**	**Friedman p=0.00*** **
**14,15-DHET**	**0.33 ± 0.1**	**0.22 ± 0.07**	**0.21 ± 0.08**	**0.18 ± 0.07**	**0*** **
7,8-EDP	0.65 ±0.35	0.54 ±0.25	0.58 ±0.3	0.72 ±0.52	0.17
**10,11-EDP**	**0.51 ±0.31**	**0.34 ±0.12**	**0.47 ±0.24**	**0.58 ±0.36**	**Friedman p=0.03***
**13,14-EDP**	**0.56 ±0.29**	**0.3 ±0.13**	**0.45 ±0.22**	**0.56 ±0.32**	**Friedman p=0.00*** **
16,17-EDP	1.28 ± 0.36	0.99 ± 0.39	0.92 ± 0.32	1.16 ± 0.55	0.12
19,20-EDP	1.16 ±0.36	1.05 ±0.34	1.25 ±0.45	1.3 ±0.52	0.3
**7,8-DiHDPA**	**0.59 ±0.36**	**0.41 ±0.15**	**0.45 ±0.22**	**0.46 ±0.26**	**Friedman p=0.02***
**10,11-DiHDPA**	**0.06 ±0.03**	**0.04 ±0.02**	**0.05 ±0.01**	**0.04 ±0.03**	**Friedman p=0.04***
**13,14-DiHDPA**	**0.1 ±0.03**	**0.08 ±0.02**	**0.08 ±0.02**	**0.07 ±0.03**	**Friedman p=0.00*** **
16,17-DiHDPA	0.19 ± 0.05	0.15 ± 0.04	0.15 ± 0.04	0.15 ± 0.07	0.05
**19,20-DiHDPA**	**2.47 ±1.28**	**1.52 ±0.56**	**1.33 ±0.77**	**1.16 ±0.88**	**Friedman p=0.00*** **
**5,6-EEQ**	**3.41 ±1.64**	**2.36 ±1.24**	**2.44 ±1.09**	**3.05 ±1.43**	**Friedman p=0.00*** **

8,9-EEQ	0.52 ±0.16	0.48 ±0.22	0.52 ±0.27	0.62 ±0.21	0.34
11,12-EEQ	0.37 ± 0.14	0.34 ± 0.17	0.38 ± 0.2	0.46 ± 0.2	0.11
14,15-EEQ	0.22 ±0.1	0.21 ±0.09	0.23 ±0.09	0.27 ±0.12	0.29
17,18-EEQ	0.61 ± 0.24	0.55 ± 0.24	0.61 ± 0.26	0.70 ± 0.22	0.08
5,6-DiHETE	0.86 ±0.47	0.85 ±0.6	0.88 ±0.48	1.04 ±0.54	0.05
8,9-DiHETE	0.07 ± 0.02	0.06 ± 0.02	0.07 ± 0.02	0.06 ± 0.03	0.21
11,12-DiHETE	0.03 ±0.01	0.03 ±0.01	0.03 ±0.01	0.03 ±0.01	0.59
**14,15-DiHETE**	**0.06 ± 0.02**	**0.05 ± 0.01**	**0.05 ± 0.01**	**0.05 ± 0.02**	**0.01****
**17,18-DiHETE**	**0.42 ±0.21**	**0.29 ±0.07**	**0.28 ±0.13**	**0.21 ±0.13**	**Friedman p=0.00*****
**B: LOX/CYP ω/(ω-1)-hydroxylase oxylipins**					
9-HODE	27.1 ±7.17	26.53 ±8.55	26.73 ±9.47	31.07 ±14.79	0.68
13-HODE	19.66 ±5.12	20.35 ±7.6	21.11 ±8.7	24.82 ±14.65	0.99
5-HETE	7.96 ±2.09	6.58 ±1.89	7.29 ±2.81	8.65 ±4	0.14
8-HETE	4.27 ±0.91	3.45 ±1.08	3.59 ±1.22	4.21 ±1.77	0.06
9-HETE	6.43 ±1.67	5.05 ±2.14	5.51 ±2.56	7.05 ±3.64	0.09
11-HETE	5.71 ±1.2	4.74 ±1.57	5.05 ±1.73	6.14 ±2.76	0.26
**12-HETE**	**6.38 ± 1.46**	**5.34 ± 1.87**	**6.36 ± 2.05**	**8.69 ± 3.44**	**0*****
**15-HETE**	**9.49 ±2.3**	**7.28 ±2.47**	**7.91 ±2.2**	**9.54 ±4.31**	**Friedman p=0.03***
**16-HETE**	**0.26 ±0.08**	**0.19 ±0.04**	**0.22 ±0.04**	**0.24 ±0.07**	**Friedman p=0.01****
**17-HETE**	**0.07 ±0.01**	**0.05 ±0.01**	**0.06 ±0.02**	**0.05 ±0.02**	**Friedman p=0.00*****
**18-HETE**	**0.16 ± 0.05**	**0.11 ± 0.04**	**0.11 ± 0.03**	**0.1 ± 0.04**	**0*****
19-HETE	0.14 ±0.04	0.11 ±0.04	0.12 ±0.05	0.12 ±0.04	0.21
20-HETE	0.67 ±0.2	0.66 ±0.22	0.86 ±0.41	0.78 ±0.4	0.19
**4-HDHA**	**1.95 ±0.59**	**1.45 ±0.47**	**1.72 ±0.51**	**2.07 ±0.92**	**Friedman p=0.00*****
**7-HDHA**	**1.62 ± 0.47**	**1.24 ± 0.42**	**1.32 ± 0.53**	**1.59 ± 0.79**	**0.04**
8-HDHA	1.45 ±0.56	1.11 ±0.49	1.15 ±0.62	1.35 ±0.8	0.07
10-HDHA	1.04 ±0.35	0.84 ±0.32	0.87 ±0.34	1.04 ±0.55	0.05
11-HDHA	1.71 ±0.62	1.32 ±0.61	1.38 ±0.63	1.64 ±0.94	0.05

**13-HDHA**	**1.26 ± 0.41**	**0.93 ± 0.39**	**0.97 ± 0.32**	**1.26 ± 0.68**	**0.04***
14-HDHA	1.33 ± 0.47	1.13 ± 0.44	1.22 ± 0.39	1.39 ± 0.67	0.2
16-HDHA	0.99 ±0.32	0.74 ±0.34	0.77 ±0.25	0.96 ±0.57	0.22
17-HDHA	2.95 ±0.88	2.43 ±0.99	2.4 ±0.85	3.1 ±1.7	0.17
20-HDHA	3.49 ±1.29	2.81 ±1.01	2.9 ±0.97	3.32 ±1.73	0.08
22-HDHA	0.23 ±0.1	0.23 ±0.14	0.29 ±0.16	0.27 ±0.2	0.34
**5-HEPE**	**0.89 ±0.44**	**0.71 ±0.34**	**0.71 ±0.38**	**0.83 ±0.52**	**Friedman p=0.00*** **
**8-HEPE**	**0.32 ±0.19**	**0.3 ±0.18**	**0.23 ±0.19**	**0.26 ±0.22**	**0.03***
9-HEPE	0.66 ±0.42	0.62 ±0.44	0.46 ±0.33	0.56 ±0.54	0.08
**11-HEPE**	**0.93 ±0.86**	**0.81 ±0.51**	**0.55 ±0.37**	**0.63 ±0.51**	**Friedman p=0.04***
12-HEPE	0.7 ±0.4	0.7 ±0.47	0.58 ±0.5	0.7 ±0.62	0.61
15-HEPE	0.56 ±0.38	0.47 ±0.28	0.37 ±0.28	0.44 ±0.38	0.06
18-HEPE	1.42 ±0.7	1.22 ±0.54	1.13 ±0.71	1.34 ±0.88	0.27
20-HEPE	0.32 ±0.11	0.3 ±0.11	0.36 ±0.16	0.34 ±0.2	0.3

Data presented as Mean ± SD. Oxylipins marked in bold black indicate statistical significance. "*" means p<0.05, "**" means p<0.01, "***" means p<0.001.

Table S4 Post-hoc comparison of paired differences in venous plasma oxylipin levels.

Units ng/ml		Tukey's post hoc test, P value
**A: CYP 450-epoxy oxlipins**		
11,12-EET	Pre-CPB-15min after CPB starts	0.21
	Pre-CPB-45min after CPB starts	0.77
	Pre-CPB-60min after CPB ends	0.43
	15min after CPB starts-45min after CPB starts	0.73
	**15min after CPB starts-60min after CPB ends**	**0.01****
	45min after CPB starts-60min after CPB ends	0.08
8,9-DHET	**Pre-CPB-15min after CPB starts**	**0.00*****
	Pre-CPB-45min after CPB starts	0.23
	Pre-CPB-60min after CPB ends	0.17
	15min after CPB starts-45min after CPB starts	0.30
	15min after CPB starts-60min after CPB ends	0.39
	45min after CPB starts-60min after CPB ends	0.90
11,12-DHET	**Pre-CPB-15min after CPB starts**	**0.01****
	**Pre-CPB-45min after CPB starts**	**0.02***
	Pre-CPB-60min after CPB ends	0.12
	15min after CPB starts-45min after CPB starts	0.90
	15min after CPB starts-60min after CPB ends	0.75
	45min after CPB starts-60min after CPB ends	0.90
14,15-DHET	**Pre-CPB-15min after CPB starts**	**0.00*****
	**Pre-CPB-45min after CPB starts**	**0.00*****
	**Pre-CPB-60min after CPB ends**	**0.00*****
	15min after CPB starts-45min after CPB starts	0.94
	15min after CPB starts-60min after CPB ends	0.34
	45min after CPB starts-60min after CPB ends	0.67
10,11-EDP	Pre-CPB-15min after CPB starts	0.17
	Pre-CPB-45min after CPB starts	0.90

	Pre-CPB-60min after CPB ends	0.84
	15min after CPB starts-45min after CPB starts	0.17
	**15min after CPB starts-60min after CPB ends**	**0.02***
	45min after CPB starts-60min after CPB ends	0.84
13,14-EDP	**Pre-CPB-15min after CPB starts**	**0.00*****
	Pre-CPB-45min after CPB starts	0.58
	Pre-CPB-60min after CPB ends	0.90
	15min after CPB starts-45min after CPB starts	0.08
	**15min after CPB starts-60min after CPB ends**	**0.00*****
	45min after CPB starts-60min after CPB ends	0.66
7,8-DiHDPA	**Pre-CPB-15min after CPB starts**	**0.04***
	Pre-CPB-45min after CPB starts	0.12
	**Pre-CPB-60min after CPB ends**	**0.04***
	15min after CPB starts-45min after CPB starts	0.90
	15min after CPB starts-60min after CPB ends	0.90
	45min after CPB starts-60min after CPB ends	0.90
10,11-DiHDPA	**Pre-CPB-15min after CPB starts**	**0.04***
	Pre-CPB-45min after CPB starts	0.90
	Pre-CPB-60min after CPB ends	0.30
	15min after CPB starts-45min after CPB starts	0.17
	15min after CPB starts-60min after CPB ends	0.75
	45min after CPB starts-60min after CPB ends	0.66
13,14-DiHDPA	Pre-CPB-15min after CPB starts	0.12
	**Pre-CPB-45min after CPB starts**	**0.01****
	**Pre-CPB-60min after CPB ends**	**0.01****
	15min after CPB starts-45min after CPB starts	0.84
	15min after CPB starts-60min after CPB ends	0.84
	45min after CPB starts-60min after CPB ends	0.90
19,20-DiHDPA	Pre-CPB-15min after CPB starts	0.12
	**Pre-CPB-45min after CPB starts**	**0.02***

	**Pre-CPB-60min after CPB ends**	**0.00*** **
	15min after CPB starts-45min after CPB starts	0.90
	15min after CPB starts-60min after CPB ends	0.39
	45min after CPB starts-60min after CPB ends	0.75
5,6-EEQ	**Pre-CPB-15min after CPB starts**	**0.00*** **
	**Pre-CPB-45min after CPB starts**	**0.01** **
	Pre-CPB-60min after CPB ends	0.39
	15min after CPB starts-45min after CPB starts	0.90
	15min after CPB starts-60min after CPB ends	0.23
	45min after CPB starts-60min after CPB ends	0.39
14,15-DiHETE	Pre-CPB-15min after CPB starts	0.16
	Pre-CPB-45min after CPB starts	0.06
	**Pre-CPB-60min after CPB ends**	**0.01** **
	15min after CPB starts-45min after CPB starts	0.97
	15min after CPB starts-60min after CPB ends	0.70
	45min after CPB starts-60min after CPB ends	0.92
17,18-DiHETE	Pre-CPB-15min after CPB starts	0.49
	**Pre-CPB-45min after CPB starts**	**0.04***
	**Pre-CPB-60min after CPB ends**	**0.00*** **
	15min after CPB starts-45min after CPB starts	0.58
	**15min after CPB starts-60min after CPB ends**	**0.04***
	45min after CPB starts-60min after CPB ends	0.49
**B: LOX/CYP ω/(ω-1)-hydroxylase oxylipins**		
12-HETE	Pre-CPB-15min after CPB starts	0.47
	Pre-CPB-45min after CPB starts	1.00
	**Pre-CPB-60min after CPB ends**	**0.01** **
	15min after CPB starts-45min after CPB starts	0.48
	**15min after CPB starts-60min after CPB ends**	**0.00*** **
	**45min after CPB starts-60min after CPB ends**	**0.01** **
15-HETE	**Pre-CPB-15min after CPB starts**	**0.01** **

	Pre-CPB-45min after CPB starts	0.30
	Pre-CPB-60min after CPB ends	0.39
	15min after CPB starts-45min after CPB starts	0.58
	15min after CPB starts-60min after CPB ends	0.49
	45min after CPB starts-60min after CPB ends	0.90
16-HETE	**Pre-CPB-15min after CPB starts**	**0.01****
	Pre-CPB-45min after CPB starts	0.75
	Pre-CPB-60min after CPB ends	0.90
	15min after CPB starts-45min after CPB starts	0.17
	15min after CPB starts-60min after CPB ends	0.06
	45min after CPB starts-60min after CPB ends	0.90
17-HETE	**Pre-CPB-15min after CPB starts**	**0.01****
	Pre-CPB-45min after CPB starts	0.58
	**Pre-CPB-60min after CPB ends**	**0.04***
	15min after CPB starts-45min after CPB starts	0.30
	15min after CPB starts-60min after CPB ends	0.90
	45min after CPB starts-60min after CPB ends	0.49
18-HETE	**Pre-CPB-15min after CPB starts**	**0.00*****
	**Pre-CPB-45min after CPB starts**	**0.01****
	**Pre-CPB-60min after CPB ends**	**0.00*****
	15min after CPB starts-45min after CPB starts	1.00
	15min after CPB starts-60min after CPB ends	0.99
	45min after CPB starts-60min after CPB ends	0.98
4-HDHA	**Pre-CPB-15min after CPB starts**	**0.01****
	Pre-CPB-45min after CPB starts	0.58
	Pre-CPB-60min after CPB ends	0.90
	15min after CPB starts-45min after CPB starts	0.30
	**15min after CPB starts-60min after CPB ends**	**0.02***
	45min after CPB starts-60min after CPB ends	0.66
7-HDHA	Pre-CPB-15min after CPB starts	0.05

	Pre-CPB-45min after CPB starts	0.19
	Pre-CPB-60min after CPB ends	1.00
	15min after CPB starts-45min after CPB starts	0.92
	15min after CPB starts-60min after CPB ends	0.08
	45min after CPB starts-60min after CPB ends	0.28
5-HEPE	**Pre-CPB-15min after CPB starts**	**0.01****
	**Pre-CPB-45min after CPB starts**	**0.02***
	Pre-CPB-60min after CPB ends	0.30
	15min after CPB starts-45min after CPB starts	0.90
	15min after CPB starts-60min after CPB ends	0.58
	45min after CPB starts-60min after CPB ends	0.66
11-HEPE	Pre-CPB-15min after CPB starts	0.75
	**Pre-CPB-45min after CPB starts**	**0.04***
	Pre-CPB-60min after CPB ends	0.17
	15min after CPB starts-45min after CPB starts	0.30
	15min after CPB starts-60min after CPB ends	0.66
	45min after CPB starts-60min after CPB ends	0.90

"*" means $p<0.05$, "**" means $p<0.01$, "***" means $p<0.001$.

Table S5 Time-profiles throughout cardiopulmonary bypass surgery with changes in expression levels of venous erythrocytes oxylipins.

Units ng/g	Time point 1: Pre-CPB	Time point 2: 15 min after CPB starts	Time point 3: 45 min after CPB starts	Time point 4: 60 min after CPB ends	Mauchly's test/Greenhouse–Geisser, P value
**A: CYP 450-epoxy oxylipins**					
9,10-EpOME	33.51±23.06	35.49±29.86	27.74±13.34	46.78 ±51.85	0.72
12,13-EpOME	34.94±20.74	37.36±26.42	30.46±12.25	49.31 ±53.14	0.65
9,10-DiHOME	3.64±1.96	3.31±1.04	3.3±1.78	10.28 ±23.72	0.47
12,13-DiHOME	6.35±2.09	6.2±1.29	6.16±2.47	15.63 ±32.89	0.55
5,6-EET	38.67±43.57	41.12±43.23	31.79±29.87	37.47 ±42.06	0.29
8,9-EET	188.49±182.2	166.53±183.22	192.44±216.85	160.13 ±158.46	0.34
11,12-EET	103.14±95.34	104.07±124.31	105.06±111.9	91.03 ±80.49	0.29
14,15-EET	147.87±120.56	129.12±110.3	151.53±147.84	133.83 ±112.46	0.47
5,6-DHET	13.91±8.84	15.21±12.38	13.56±5.9	12.19 ±5.31	0.37
8,9-DHET	2.1±0.49	2.09±0.5	2.3±0.59	2.27 ±0.57	0.4
11,12-DHET	1.74±0.52	2.03±0.82	1.8±0.56	1.89 ±0.65	0.63
14,15-DHET	0.93±0.23	1.14±0.35	1.13±0.43	1.17 ±0.58	0.51
7,8-EDP	103.85±102.59	83.19±92.86	92.98±113.93	78.27 ±80.63	0.34
10,11-EDP	44.88±41.49	36.07±34.74	41.41±47.74	36.84 ±35.77	0.75
13,14-EDP	30.94±25.88	23.84±19.9	28.19±29.87	24.97 ±22.67	0.87
16,17-EDP	12.39±9.59	11.12±8.84	10.2±7.97	10.43 ±7.82	0.82
19,20-EDP	4.57±3.25	5.33±3.47	5.25±3.82	5.33 ±4.04	0.18
7,8-DiHDPA	2.64±1.39	2.16±0.87	2.59±1.32	2.8 ±1.42	0.02
10,11-DiHDPA	0.36±0.09	0.39±0.15	0.37±0.09	0.38 ±0.13	0.77
13,14-DiHDPA	0.15±0.09	0.21±0.25	0.17±0.11	0.14 ±0.1	0.72

16,17-DiHDPA	0.24±0.12	0.27±0.129	0.26±0.14	0.27 ±0.16	0.77
19,20-DiHDPA	1.02±0.78	1.21±0.91	1.74±2.55	1.03 ±0.73	0.06
5,6-EEQ	28.49±28.62	28.26±25.64	17.42±10.18	24.1 ±30.34	0.47
8,9-EEQ	3.34±1.09	3.1±1.17	2.97±1.49	2.96 ±1.22	0.68
11,12-EEQ	2.64 ± 0.72	2.42 ± 0.84	2.38 ± 1.05	2.42 ± 0.94	0.63
14,15-EEQ	1.85±0.64	1.8±0.99	1.84±0.89	1.73 ±0.72	0.37
17,18-EEQ	2±1.38	2.15±1.54	2.06±1.75	2.02 ±1.46	0.59
5,6-DiHETE	4.39±3.9	6.08±5.75	4.65±4.8	4.93 ±5.5	0.37
8,9-DiHETE	0.14 ± 0.04	0.14 ± 0.04	0.14 ± 0.05	0.14 ± 0.03	0.76
11,12-DiHETE	0.11±0.09	0.17±0.21	0.13±0.14	0.11 ±0.1	0.87
17,18-DiHETE	0.15 ± 0.11	0.16 ± 0.10	0.15 ± 0.08	0.14 ± 0.08	0.32
**B: LOX/CYP ω/(ω-1)-hydroxylase oxylipins**					
9-HODE	82.59±45.05	120.21±127.37	97.25±61.1	88.99 ±69.16	0.59
13-HODE	22.25±13.85	50.31±109.21	25.71±23.56	35.18 ±49.74	0.59
5-HETE	72.91±51.69	130.17±216.18	68.34±40.68	64.67 ±37.59	0.82
8-HETE	44.96±29.8	57.55±53.59	46.06±24.98	40.19 ±20.13	0.19
9-HETE	58.61±36.8	95.13±115.95	58.62±34.22	54.1 ±31.11	0.44
11-HETE	58.22±35.5	77.15±76.39	58.9±30.92	52.58 ±25.65	0.24
12-HETE	203.75±127.02	225.72±215.49	194.91±135.72	182.93 ±105.68	0.47
15-HETE	141.39±86.87	208.84±235.55	148.08±82.04	129.23 ±75.04	0.44
16-HETE	3.15±2.1	5.03±6.18	3.43±2	3.08 ±2.17	0.17
17-HETE	0.09±0.06	0.08±0.03	0.08±0.02	0.08 ±0.03	0.75
18-HETE	0.24 ± 0.15	0.26 ± 0.13	0.27 ± 0.12	0.25 ± 0.13	0.66
19-HETE	0.57±0.29	0.57±0.27	0.65±0.39	0.51 ±0.19	0.59

4-HDHA	28.77±18.91	46.87±72.49	26.18±16.17	24.33 ±14.6	0.51
7-HDHA	9.84±4.94	12.96±11.2	9.28±5.17	8.72 ±3.91	0.27
8-HDHA	8.94±5.23	14.42±12.75	11.29±7.52	9.94 ±7.3	0.4
10-HDHA	9.07±5.05	10.54±8.87	8.53±4.9	7.64 ±3.67	0.34
11-HDHA	20.7±12.21	24.2±21.35	19.9±12	17.51 ±8.98	0.75
13-HDHA	18.11±8.18	20.29±13.11	17±8.15	15.91 ±5.92	0.82
14-HDHA	16.29±8.04	19.03±15.73	15.47±8.58	13.96 ±6.91	0.37
16-HDHA	13.97±5.75	17.17±10.4	14.2±7	13.66 ±6.99	0.23
17-HDHA	16.67±8.41	21.44±15.03	18.03±11.06	16.62 ±9.98	0.37
20-HDHA	26.57±19.84	35.48±30.98	28.48±22.92	25.89 ±21.88	0.12
5-HEPE	6.75±4.03	8.8±10.88	6.02±4.61	5.46 ±4.04	0.47
8-HEPE	1.44±0.86	1.67±1.28	1.32±0.95	1.19 ±0.85	0.15
9-HEPE	4.21±2.76	5.2±4.88	3.93±3.06	3.4 ±2.72	0.45
11-HEPE	1.42±0.86	1.74±1.48	1.34±0.95	1.19 ±0.9	0.34
12-HEPE	8.59 ± 4.68	11 ± 7.2	9.65 ± 6.7	8.6 ± 5.49	0.47
15-HEPE	3.04±2.14	3.62±2.86	2.98±2.57	2.77 ±2.43	0.26
18-HEPE	5.13±7.81	6.47±7.89	5.35±7.52	5.16 ±7.92	0.12

Data presented as Mean ± SD.

Table S6 Time-profiles throughout cardiopulmonary bypass surgery with changes in expression levels of arterial plasma oxylipins.

Units ng/ml	Time point 1: Pre-CPB	Time point 2: 15 min after CPB starts	Time point 3: 45 min after CPB starts	Time point 4: 60 min after CPB ends	Mauchly's test/Greenhouse–Geisser, P value
9,10-EpOME	8.97 ±3.39	8.94 ±2.35	10.45 ±4.36	9.66 ±2.49	0.15
12,13-EpOME	9.27 ±3.54	9.18 ±2.15	10.4±3.4	9.7 ±2.16	0.59
9,10-DiHOME	18.11 ±17.98	21.04 ±9.35	21.85 ±11.58	21.42 ±9.32	0.47
12,13-DiHOME	17.13 ±14.4	18.74 ±6.76	19.7 ±9.82	17.59 ±6.2	0.42
5,6-EET	0.03 ±0.02	0.03 ±0.03	0.02 ±0.01	0.03 ±0.01	0.43
8,9-EET	4.2 ± 1.8	3.86 ± 1.27	3.97 ± 1.83	3.92 ± 1.35	0.67
11,12-EET	2.14 ± 2.14	1.86 ± 0.79	2.01 ± 0.86	2.05 ± 0.85	0.5
14,15-EET	5.94 ± 2.7	5.96 ± 2.52	5.51 ± 2.14	5.72 ± 1.85	0.83
**5,6-DHET**	**1.23 ±0.69**	**1.01 ±0.61**	**0.98 ±0.45**	**0.93 ±0.33**	**Friedman p=0.04***
8,9-DHET	1.42 ±0.6	1.26 ±0.5	1.32 ±0.62	1.22 ±0.52	0.1
11,12-DHET	0.58 ± 0.2	0.53 ± 0.19	0.53 ± 0.25	0.47 ± 0.16	0.11
**14,15-DHET**	**0.29 ± 0.08**	**0.24 ± 0.08**	**0.21 ± 0.11**	**0.19 ± 0.08**	**0.02***
7,8-EDP	0.76 ±0.72	0.58 ±0.32	0.65 ±0.36	0.64 ±0.34	0.47
10,11-EDP	0.65 ±0.81	0.43 ±0.24	0.51 ±0.29	0.54 ±0.31	0.15
13,14-EDP	0.65 ±0.74	0.39 ±0.27	0.55 ±0.32	0.51 ±0.24	0.08
**16,17-EDP**	**1.39 ± 0.51**	**1.04 ± 0.34**	**1.05 ± 0.22**	**1.06 ± 0.31**	**0.03***
19,20-EDP	1.32 ±0.89	1.21 ±0.36	1.31 ±0.46	1.27 ±0.6	0.32
7,8-DiHDPA	0.55 ±0.27	0.48 ±0.31	0.45 ±0.23	0.41 ±0.17	0.18
10,11-DiHDPA	0.05 ±0.03	0.04 ±0.02	0.04 ±0.01	0.04 ±0.02	0.12
**13,14-DiHDPA**	**0.1 ± 0.03**	**0.09 ± 0.03**	**0.08 ± 0.02**	**0.07 ± 0.02**	**0***

**16,17-DiHDPA**	**0.18 ±0.05**	**0.15 ±0.04**	**0.16 ±0.05**	**0.14 ±0.06**	**Friedman p=0.02***
**19,20-DiHDPA**	**2.49 ±1.21**	**1.59 ±0.56**	**1.4 ±1.05**	**1.11 ±0.88**	**Friedman p=0.00*** **
5,6-EEQ	2.93 ± 1.2	2.82 ± 0.99	2.7 ± 1.32	2.65 ± 1.05	0.77
8,9-EEQ	0.56 ± 0.28	0.49 ± 0.17	0.55 ± 0.20	0.56 ± 0.27	0.54
11,12-EEQ	0.43 ±0.23	0.34 ±0.1	0.35 ±0.17	0.47 ±0.21	0.45
14,15-EEQ	0.28 ±0.15	0.24 ±0.08	0.25 ±0.12	0.24 ±0.11	0.38
17,18-EEQ	0.69 ± 0.27	0.57 ± 0.19	0.62 ± 0.18	0.64 ± 0.2	0.14
5,6-DiHETE	0.56 ± 0.28	0.83 ± 0.39	1.03 ± 0.55	0.98 ± 0.46	0.27
8,9-DiHETE	0.08 ± 0.03	0.07 ± 0.02	0.07 ± 0.02	0.06 ± 0.02	0.27
11,12-DiHETE	0.03 ± 0.01	0.03 ± 0.01	0.03 ± 0.01	0.03 ± 0.01	0.37
**14,15-DiHETE**	**0.07 ± 0.02**	**0.05 ± 0.01**	**0.05 ± 0.02**	**0.04 ± 0.01**	**0*** **
**17,18-DiHETE**	**0.42 ±0.2**	**0.3 ±0.08**	**0.27 ±0.14**	**0.21 ±0.12**	**Friedman p=0.00*** **
9-HODE	26.06 ±6.44	30.87 ±19.87	27.94 ±11.08	28.41 ±9.05	0.51
13-HODE	19.18 ±4.86	23.84 ±16.09	21.51 ±9.2	22.55 ±8.85	0.47
5-HETE	8.21 ±1.79	7.52 ±2.49	7.88 ±2.67	8.56 ±4.51	0.18
8-HETE	4.07 ±1.08	3.93 ±1.41	4.24 ±1.93	4.54 ±2.57	0.47
9-HETE	6.07 ±1.45	5.82 ±2.41	6.38 ±3.65	7.4 ±4.6	0.57
11-HETE	5.25 ±1.3	5.39 ±1.98	5.55 ±2.59	6.49 ±3.58	0.72
**12-HETE**	**5.93 ±1.13**	**6.94 ±2.18**	**7.16 ±4.2**	**9.16 ±4.43**	**Friedman p=0.00*** **
15-HETE	8.57 ±2.25	8.19 ±3.49	7.88 ±2.5	10.09 ±5.32	0.29
16-HETE	0.26 ±0.08	0.23 ±0.09	0.22 ±0.06	0.22 ±0.05	0.09
**17-HETE**	**0.08 ± 0.02**	**0.06 ± 0.02**	**0.06 ± 0.02**	**0.05 ± 0.02**	**0*** **
**18-HETE**	**0.15 ± 0.04**	**0.13 ± 0.04**	**0.12 ± 0.04**	**0.1 ± 0.04**	**0.01** **
19-HETE	0.13 ± 0.05	0.12 ± 0.03	0.13 ± 0.06	0.12 ± 0.04	0.62

20-HETE	0.66 ±0.19	0.78 ±0.32	0.89 ±0.49	0.73 ±0.3	0.12
4-HDHA	2.17 ± 0.89	2.02 ± 0.92	1.96 ± 0.81	2.22 ± 1.14	0.4
7-HDHA	1.62 ± 0.48	1.38 ± 0.56	1.42 ± 0.68	1.62 ± 0.92	0.31
8-HDHA	1.35 ±0.5	1.29 ±0.72	1.41 ±1.17	1.58 ±1.28	0.72
10-HDHA	0.98 ±0.23	0.94 ±0.42	1.05 ±0.77	1.16 ±0.77	0.75
11-HDHA	1.61 ±0.5	1.57 ±0.79	1.61 ±1.32	1.84 ±1.5	0.34
13-HDHA	1.12 ±0.36	1.12 ±0.57	1.09 ±0.79	1.22 ±0.8	0.47
14-HDHA	1.29 ±0.29	1.31 ±0.59	1.34 ±0.85	1.66 ±1.02	0.24
**16-HDHA**	**0.99 ±0.28**	**0.8 ±0.36**	**0.82 ±0.5**	**0.97 ±0.62**	**Friedman p=0.00*****
17-HDHA	2.67 ±0.7	2.7 ±1.19	2.84 ±1.63	3.31 ±2.23	0.61
20-HDHA	3.24 ±1.06	3.09 ±1.34	2.96 ±1.15	3.55 ±1.84	0.29
22-HDHA	0.2 ±0.07	0.32 ±0.3	0.3 ±0.2	0.24 ±0.16	0.48
5-HEPE	1.04 ±0.56	0.79 ±0.49	0.76 ±0.52	0.86 ±0.63	0.06
**8-HEPE**	**0.34 ±0.14**	**0.27 ±0.19**	**0.26 ±0.3**	**0.3 ±0.32**	**Friedman p=0.00****
**9-HEPE**	**0.74 ±0.35**	**0.58 ±0.43**	**0.55 ±0.65**	**0.64 ±0.74**	**Friedman p=0.04***
**11-HEPE**	**0.97 ±0.55**	**0.68 ±0.47**	**0.58 ±0.5**	**0.68 ±0.62**	**Friedman p=0.00****
12-HEPE	0.79 ±0.33	0.66 ±0.52	0.68 ±0.77	0.81 ±0.96	0.45
**15-HEPE**	**0.58 ±0.25**	**0.42 ±0.29**	**0.41 ±0.42**	**0.5 ±0.54**	**Friedman p=0.03***
18-HEPE	1.42 ±0.55	1.15 ±0.67	1.14 ±0.72	1.4 ±0.96	0.21
20-HEPE	0.31 ± 0.11	0.35 ± 0.13	0.35 ± 0.13	0.33 ± 0.17	0.68

Data presented as Mean ± SD. Oxylipins marked in bold black indicate statistical significance. "*" means p<0.05, "**" means p<0.01, "***" means p<0.001.

Table S7 Post-hoc comparison of paired differences in arterial plasma oxylipin levels.

Units ng/ml		Tukey's post hoc test, P value
**A: CYP 450-epoxy oxylipins**		
5,6-DHET	**Pre-CPB-15min after CPB starts**	**0.04***
	Pre-CPB-45min after CPB starts	0.66
	Pre-CPB-60min after CPB ends	0.23
	15min after CPB starts-45min after CPB starts	0.39
	15min after CPB starts-60min after CPB ends	0.84
	45min after CPB starts-60min after CPB ends	0.84
14,15-DHET	Pre-CPB-15min after CPB starts	0.42
	**Pre-CPB-45min after CPB starts**	**0.04***
	**Pre-CPB-60min after CPB ends**	**0.01****
	15min after CPB starts-45min after CPB starts	0.62
	15min after CPB starts-60min after CPB ends	0.27
	45min after CPB starts-60min after CPB ends	0.92
13,14-DiHDPA	Pre-CPB-15min after CPB starts	0.34
	Pre-CPB-45min after CPB starts	0.32
	**Pre-CPB-60min after CPB ends**	**0.03***
	15min after CPB starts-45min after CPB starts	0.98
	15min after CPB starts-60min after CPB ends	0.87
	45min after CPB starts-60min after CPB ends	0.68
16,17-DiHDPA	**Pre-CPB-15min after CPB starts**	**0.04***
	Pre-CPB-45min after CPB starts	0.39
	**Pre-CPB-60min after CPB ends**	**0.04***
	15min after CPB starts-45min after CPB starts	0.66
	15min after CPB starts-60min after CPB ends	0.90
	45min after CPB starts-60min after CPB ends	0.66
19,20-DiHDPA	Pre-CPB-15min after CPB starts	0.58
	**Pre-CPB-45min after CPB starts**	**0.01****
	**Pre-CPB-60min after CPB ends**	**0.00*****
	15min after CPB starts-45min after CPB starts	0.23
	15min after CPB starts-60min after CPB ends	0.06
	45min after CPB starts-60min after CPB ends	0.90
14,15-DiHETE	**Pre-CPB-15min after CPB starts**	**0.03***
	**Pre-CPB-45min after CPB starts**	**0.04***
	**Pre-CPB-60min after CPB ends**	**0.00*****
	15min after CPB starts-45min after CPB starts	1.00
	15min after CPB starts-60min after CPB ends	0.54
	45min after CPB starts-60min after CPB ends	0.50
17,18-DiHETE	Pre-CPB-15min after CPB starts	0.30
	**Pre-CPB-45min after CPB starts**	**0.04***

	**Pre-CPB-60min after CPB ends**	**0.00*** **
	15min after CPB starts-45min after CPB starts	0.75
	15min after CPB starts-60min after CPB ends	0.17
	45min after CPB starts-60min after CPB ends	0.66
**B: LOX/CYP ω/(ω-1)-hydroxylase oxylipins**		
12-HETE	Pre-CPB-15min after CPB starts	0.39
	Pre-CPB-45min after CPB starts	0.90
	**Pre-CPB-60min after CPB ends**	**0.01** **
	15min after CPB starts-45min after CPB starts	0.58
	15min after CPB starts-60min after CPB ends	0.39
	**45min after CPB starts-60min after CPB ends**	**0.02***
17-HETE	Pre-CPB-15min after CPB starts	0.09
	**Pre-CPB-45min after CPB starts**	**0.04***
	**Pre-CPB-60min after CPB ends**	**0.00*** **
	15min after CPB starts-45min after CPB starts	0.98
	15min after CPB starts-60min after CPB ends	0.20
	45min after CPB starts-60min after CPB ends	0.40
18-HETE	Pre-CPB-15min after CPB starts	0.19
	**Pre-CPB-45min after CPB starts**	**0.04***
	**Pre-CPB-60min after CPB ends**	**0.01** **
	15min after CPB starts-45min after CPB starts	0.89
	15min after CPB starts-60min after CPB ends	0.41
	45min after CPB starts-60min after CPB ends	0.82
16-HDHA	**Pre-CPB-15min after CPB starts**	**0.04***
	**Pre-CPB-45min after CPB starts**	**0.01** **
	Pre-CPB-60min after CPB ends	0.30
	15min after CPB starts-45min after CPB starts	0.90
	15min after CPB starts-60min after CPB ends	0.75
	45min after CPB starts-60min after CPB ends	0.49
8-HEPE	Pre-CPB-15min after CPB starts	0.12
	**Pre-CPB-45min after CPB starts**	**0.01** **
	Pre-CPB-60min after CPB ends	0.17
	15min after CPB starts-45min after CPB starts	0.66
	15min after CPB starts-60min after CPB ends	0.90
	45min after CPB starts-60min after CPB ends	0.58
9-HEPE	Pre-CPB-15min after CPB starts	0.90
	**Pre-CPB-45min after CPB starts**	**0.04***
	Pre-CPB-60min after CPB ends	0.30
	15min after CPB starts-45min after CPB starts	0.17
	15min after CPB starts-60min after CPB ends	0.66
	45min after CPB starts-60min after CPB ends	0.75
11-HEPE	Pre-CPB-15min after CPB starts	0.49
	**Pre-CPB-45min after CPB starts**	**0.00*** **

	Pre-CPB-60min after CPB ends	0.06
	15min after CPB starts-45min after CPB starts	0.12
	15min after CPB starts-60min after CPB ends	0.66
	45min after CPB starts-60min after CPB ends	0.66
15-HEPE	Pre-CPB-15min after CPB starts	0.30
	**Pre-CPB-45min after CPB starts**	**0.01****
	Pre-CPB-60min after CPB ends	0.39
	15min after CPB starts-45min after CPB starts	0.58
	15min after CPB starts-60min after CPB ends	0.90
	45min after CPB starts-60min after CPB ends	0.49

"*" means p<0.05, "**" means p<0.01, "***" means p<0.001.

Table S8 Time-profiles throughout cardiopulmonary bypass surgery with changes in expression levels of arterial erythrocytes oxylipins.

Units ng/g	Time point 1: Pre-CPB	Time point 2: 15 min after CPB starts	Time point 3: 45 min after CPB starts	Time point 4: 60 min after CPB ends	Mauchly's test/Greenhouse–Geisser, P value
9,10-EpOME	76.61 ±102.53	69.03 ±84.47	49.73 ±45.62	43.02 ±36.37	0.11
12,13-EpOME	74.44 ±87.5	69.51 ±72.47	52.64 ±45.41	45.22 ±37.11	0.12
9,10-DiHOME	4.12 ±2.96	4.4 ±2.63	3.61 ±1.56	3.54 ±1.59	0.68
12,13-DiHOME	7.89 ±4.38	7.97 ±3.6	6.98 ±2.3	6.69 ±2.83	0.24
5,6-EET	45.78 ±49.55	38.78 ±42.92	39.11 ±47.14	31.48 ±35.77	0.73
8,9-EET	222.15 ±217.64	164.11 ±174.99	226.9 ±287.08	176.84±184.57	0.30
11,12-EET	154.54 ±174.47	114.23 ±125.53	131.32 ±149.61	103.26 ±96.28	0.90
14,15-EET	157.79 ±132.74	123.24 ±107.7	166.95 ±188.11	134.65 ±118.82	0.46
5,6-DHET	20.33 ±15.51	18.8 ±12.77	20.51 ±13.03	17.42 ±12.02	0.44
8,9-DHET	2.34 ±0.86	2.18 ±0.37	2.27 ±0.46	2.43 ±1.08	0.64
**11,12-DHET**	**3.77 ±3.59**	**3.19 ±2.6**	**2.85 ±2.11**	**2.89 ±2.2**	**Friedman p=0.04***
14,15-DHET	1.95 ±1.99	1.49 ±0.81	1.47 ±0.69	1.76 ±1.86	0.22
7,8-EDP	113.77 ±111.18	80.81 ±87.41	116.22 ±155.49	85.68 ±91.26	0.44
10,11-EDP	47.36 ±45.08	33.41 ±34.11	49.27 ±66.21	37.62 ±38.18	0.55
13,14-EDP	28.6 ±25.73	21.53 ±19.87	30.64 ±37.83	24.2 ±22.82	0.24
16,17-EDP	13.44 ±10.77	10.97 ±8	13.85 ±13.44	9.88 ±7.23	0.06
19,20-EDP	4.62 ±3.15	5.45 ±3.78	5.73 ±3.87	4.95 ±3.52	0.325
7,8-DiHDPA	2.53 ±1.34	2.19 ±0.89	2.49 ±1.12	2.74 ±1.4	0.30
10,11-DiHDPA	0.62 ±0.52	0.58 ±0.43	0.54 ±0.33	0.6 ±0.47	0.12
13,14-DiHDPA	0.52 ±0.75	0.62 ±0.93	0.59 ±0.96	0.51 ±0.89	0.44
16,17-DiHDPA	0.39 ±0.35	0.39 ±0.26	0.39 ±0.26	0.45 ±0.4	0.28
**19,20-DiHDPA**	**2.26 ±3.3**	**2.14 ±2.71**	**2.07 ±2.49**	**2 ±2.41**	**Friedman p=0.01****

5,6-EEQ	31.48 ±36.04	21.82 ±17.7	20.84 ±20.93	21.8 ±27.3	0.30
8,9-EEQ	2.91 ±1.11	2.76 ±0.96	3.04 ±1.26	3.1 ±1.31	0.556
11,12-EEQ	2.42 ±0.88	2.23 ±0.76	2.39 ±0.73	2.35 ±0.78	0.802
14,15-EEQ	1.63 ±0.43	1.62 ±0.53	1.8 ±0.5	1.63 ±0.45	0.509
17,18-EEQ	1.66 ±0.83	1.83 ±0.99	2 ±1.01	1.83 ±0.9	0.164
5,6-DiHETE	7.57 ±7.18	8.88 ±9.28	7.76 ±5.89	8.73 ±11.55	0.12
8,9-DiHETE	0.18 ±0.08	0.17 ±0.07	0.18 ±0.06	0.17 ±0.09	0.64
11,12-DiHETE	0.54 ±0.96	0.49 ±0.99	0.48 ±0.94	0.19 ±0.22	0.53
17,18-DiHETE	0.18±0.15	0.16 ±0.09	0.16 ±0.08	0.17 ±0.14	0.14
**9-HODE**	**243.31 ±279.8**	**236.12 ±234.18**	**231.19 ±223.68**	**170.68 ±182.99**	**Friedman p=0.01****
**13-HODE**	**181.19 ±320.76**	**137.45 ±225.91**	**108.23 ±162.21**	**80.5 ±118.6**	**Friedman p=0.01****
5-HETE	285.09 ±430.66	287.77 ±501.57	212.71 ±232.09	156.82 ±177.79	0.08
8-HETE	77.61 ±61.24	80.81 ±71.23	81.03 ±65.84	69.41 ±56.29	0.16
**9-HETE**	**181.34 ±196.26**	**195.24 ±248.02**	**172.76 ±161.87**	**130.98 ±137.09**	**Friedman p=0.01****
11-HETE	115.12 ±95.11	119.63 ±111.21	119 ±95.85	98.77 ±82.43	0.31
12-HETE	302.73 ±183.58	303.27 ±236.77	318.97 ±257.14	242.31 ±145.11	0.98
15-HETE	342.62 ±344.23	373.66 ±407.79	336.16 ±304.37	275.68 ±288.54	0.06
16-HETE	10.07 ±12.6	10.45 ±12.94	9.52 ±11.27	7.84 ±10.75	0.19
17-HETE	0.12 ±0.1	0.09 ±0.06	0.11 ±0.08	0.11 ±0.07	0.23
18-HETE	0.27 ±0.16	0.27 ±0.14	0.29 ±0.16	0.28 ±0.14	0.875
19-HETE	0.56 ±0.32	0.68 ±0.37	0.75 ±0.5	0.66 ±0.32	0.68
**4-HDHA**	**81.59 ±103.29**	**90.34 ±147.42**	**70.04 ±71.28**	**51.24 ±54.14**	**Friedman p=0.02***
7-HDHA	18.04 ±12.71	19.36 ±18.69	18.63 ±13.85	14.86 ±11.37	0.13
8-HDHA	18.01 ±10.65	18.64 ±13.74	22.03 ±14.66	18.75 ±16.5	0.06
10-HDHA	13.45 ±8.82	13.91 ±10.42	14.73 ±11.58	12.14 ±9.31	0.44
11-HDHA	31.66 ±23.12	33.37 ±26.82	34.66 ±28.96	27.86 ±22.92	0.23

13-HDHA	24.96 ±12.83	26.15 ±15.55	27.16 ±16.58	23.24 ±13.51	0.33
14-HDHA	23.41 ±12.7	24.91 ±17.13	26.37 ±19.55	20.25 ±13.14	0.33
16-HDHA	20.09 ±8.75	23.01 ±12.08	23.21 ±12.39	20.35 ±13.81	0.06
17-HDHA	24.67 ±11.68	28.88 ±15.91	29.84 ±17.07	25.24 ±19.29	0.11
20-HDHA	46.98 ±42.25	56.09 ±48.83	55.25 ±46.9	47.45 ±54.18	0.10
5-HEPE	13.85±12.19	13.75 ±15.37	13.12 ±12.38	10.85 ±10.89	0.35
8-HEPE	2.36 ±1.42	2.22 ±1.6	2.44 ±1.83	2.09 ±1.92	0.30
**9-HEPE**	**8.04 ±5.3**	**7.79 ±6.25**	**8.53 ±6.86**	**6.91 ±6.61**	**Friedman p=0.01*** **
11-HEPE	2.6 ±1.73	2.63 ±2.06	2.76 ±2.16	2.32 ±2.11	0.09
12-HEPE	12.97 ±6.46	12.41 ±7.02	13.61 ±8.62	10.51 ±6.23	0.566
15-HEPE	5.23 ±3.46	5.46 ±4.54	5.78 ±4.43	4.8 ±4.46	0.38
18-HEPE	11.06 ±18.8	12.97 ±19.33	12.62 ±18.72	11.79 ±21.19	Friedman p=0.05

Data presented as Mean ± SD. Oxylipins marked in bold black indicate statistical significance. "*" means p<0.05, "**" means p<0.01, "***" means p<0.001.

Table S9 Post-hoc comparison of paired differences in arterial erythrocyte oxylipin levels.

Units ng/g		Tukey's post hoc test, P value
**A: CYP 450-epoxy oxylinpins**		
19,20-DiHDPA	Pre-CPB-15min after CPB starts	0.90
	Pre-CPB-45min after CPB starts	0.75
	Pre-CPB-60min after CPB ends	0.12
	15min after CPB starts-45min after CPB starts	0.75
	15min after CPB starts-60min after CPB ends	0.12
	**45min after CPB starts-60min after CPB ends**	**0.01****
**B: LOX/CYP ω/(ω-1)-hydroxylase oxylipins**		
9-HODE	Pre-CPB-15min after CPB starts	0.75
	Pre-CPB-45min after CPB starts	0.90
	Pre-CPB-60min after CPB ends	0.08
	15min after CPB starts-45min after CPB starts	0.84
	**15min after CPB starts-60min after CPB ends**	**0.01****
	45min after CPB starts-60min after CPB ends	0.06
13-HODE	Pre-CPB-15min after CPB starts	0.75
	Pre-CPB-45min after CPB starts	0.90
	Pre-CPB-60min after CPB ends	0.08
	15min after CPB starts-45min after CPB starts	0.49
	**15min after CPB starts-60min after CPB ends**	**0.01****
	45min after CPB starts-60min after CPB ends	0.23
9-HETE	Pre-CPB-15min after CPB starts	0.75
	Pre-CPB-45min after CPB starts	0.75
	Pre-CPB-60min after CPB ends	0.23
	15min after CPB starts-45min after CPB starts	0.90
	**15min after CPB starts-60min after CPB ends**	**0.02***
	**45min after CPB starts-60min after CPB ends**	**0.02***
4-HDHA	Pre-CPB-15min after CPB starts	0.90
	Pre-CPB-45min after CPB starts	0.66
	Pre-CPB-60min after CPB ends	0.23
	15min after CPB starts-45min after CPB starts	0.75
	15min after CPB starts-60min after CPB ends	0.17
	**45min after CPB starts-60min after CPB ends**	**0.01****
9-HEPE	Pre-CPB-15min after CPB starts	0.90
	Pre-CPB-45min after CPB starts	0.58
	Pre-CPB-60min after CPB ends	0.12
	15min after CPB starts-45min after CPB starts	0.39
	15min after CPB starts-60min after CPB ends	0.23
	**45min after CPB starts-60min after CPB ends**	**0.00*****

"*" means p<0.05, "**" means p<0.01, "***" means p<0.001.
